# Evaluation of the predictive and prognostic potential of blood immune cell profiles in metastatic cancer patients treated with immune checkpoint inhibitors

**DOI:** 10.1007/s00262-026-04404-0

**Published:** 2026-05-02

**Authors:** Veera Nurmela, Anna-Mari Schroderus, Satu Tiainen, Outi Kuittinen, Olli Tenhunen, Anna Jokimäki, Sanna Suoranta, Sanna Pasonen-Seppänen, Tero Sievänen, Tuure Kinnunen, Aino Rönkä

**Affiliations:** 1 Institute of Clinical Medicine, School of Medicine, University of Eastern Finland, Kuopio, Finland; 2 FICAN East, The Wellbeing Services County of North Savo, Kuopio, Finland; 3 The Wellbeing Services County of North Savo, Kuopio University Hospital, Cancer Center, Kuopio, Finland; 4 Medical Research Center Oulu and Oulu University Hospital Cancer Center, Oulu, Finland; 5 Institute of Biomedicine, School of Medicine, University of Eastern Finland, Kuopio, Finland; 6 UEF Bioinformatics Center, Institute of Biomedicine, University of Eastern Finland, Kuopio, Finland; 7 ISLAB Laboratory Center, Kuopio, Finland

**Keywords:** Peripheral blood, Cancer immunotherapy, Immune checkpoint inhibitor, Prediction, Prognosis, Biomarkers

## Abstract

Immune checkpoint inhibitors (ICI) have transformed the treatment of metastatic malignancies, yet only 20–40% of unselected patients benefit. Peripheral blood could offer a minimally invasive and dynamic source for predictive biomarkers of ICI therapy.

This study evaluated circulating immune cell frequencies and phenotypes before and during ICI therapy to identify tumor-agnostic blood-based biomarkers. We analyzed routine blood immune cell counts in a retrospective cohort of 202 patients treated with ICIs. Next, we investigated the findings in a prospectively collected cohort of 45 patients using multiparametric flow cytometry for characterization of immune cell subsets. We considered early radiological response, progression-free survival (PFS), and overall survival (OS) as clinical outcomes.

Higher pre-treatment monocyte and lower lymphocyte counts were consistently associated with inferior PFS and OS but not with early radiological response in the retrospective cohort. In the prospective cohort, detailed immunophenotyping identified elevated pre-treatment frequencies of intermediate (CD14⁺CD16⁺) monocytes as a marker of poorer PFS and OS. Within lymphocyte subsets, higher T cell frequencies were associated with better OS, and a higher pre-treatment frequency of CD226-expressing CD8⁺ memory T cells with better ICI response. ICI therapy induced increase in TIGIT^+^CD8^+^ and CD4^+^ memory T cell subsets, regardless of treatment response.

In conclusion, elevated pre-treatment levels of blood monocytes, together with decreased levels lymphocytes, were associated with poor survival of ICI-treated patients. Although detailed immunophenotyping of pre-treatment blood immune cell populations showed limited predictive utility, specific subpopulations, such as intermediate monocytes and CD226⁺CD8⁺ memory T cells, may harbor potential as prognostic/predictive indicators.

**Supplementary Information:**

The online version contains supplementary material available at 10.1007/s00262-026-04404-0.

## Introduction

Immune checkpoint inhibitors (ICIs) have emerged as the standard of care for several metastatic malignancies, with yearly expanding therapeutic indications [1]. However, only 20–40% of unselected patients benefit from the therapy, underscoring the need for predictive biomarkers [2]. Currently used biomarkers, such as PD-L1 expression or tumor mutational burden (TMB), are analyzed from tumor-tissue, and restricted access their use in clinical practice.

Peripheral blood provides a dynamic and mini-invasive source of potential biomarkers, capturing signals from tumor–immune interactions while also reflecting the overall systemic immune milieu. Since the pharmacodynamic effect of ICIs is based on reviving anti-tumor immune activity, monitoring immune states from blood may serve as a surrogate for the evaluation of ICI response. Several previous studies have demonstrated that overall frequencies of major immune cell populations exert predictive value in ICI-treated patients. For example, low baseline lymphocyte levels and elevated monocyte counts have repeatedly been linked with poorer outcomes across cancer types [3, 4]. Yet the specific immune cell subsets responsible for these associations remain incompletely defined. For example, evidence regarding the widely studied CD8⁺ T lymphocyte population is still conflicting across studies [5–7], and a similar inconsistency is seen in monocyte subtypes [8, 9]. The interpretation of these findings is also complicated by considerable heterogeneity in the endpoints and biomarker measures used across studies, with some focusing on early responses and others on long-term survival, thereby obscuring whether the findings reflect predictive or merely prognostic associations.

In addition to the counts of circulating immune cells, their functional characteristics may play a role in the antitumor response to ICIs. T cells are of particular interest in this regard since ICIs primarily target T-cell activation. During anti-PD-(L)1 therapy, exhausted T cells are functionally reinvigorated and thereby regain their capacity to mediate antitumor effects. Yet blockade of the PD-1/PD-L1 axis may also trigger compensatory upregulation of other coinhibitory receptors, such as T cell Ig and ITIM domain (TIGIT) or lymphocyte-activation gene 3 (LAG-3), which may contribute to ICI resistance, and provides a rationale for combined checkpoint blockade strategies [10, 11]. Longitudinal profiling of such T-cell subsets expressing co-inhibitory receptors during ICI therapy may therefore help to identify patient populations who could benefit from combination therapy approaches.

In the present work, we evaluated the value of routinely measured peripheral blood immune cells as therapy biomarkers in patients treated with ICIs and examined whether detailed immune phenotyping provides additional prognostic value beyond these routine blood parameters.

## Material and methods

### Study population and materials

This study included two cohorts of patients with metastatic cancers treated with ICI therapy, named retrospective and flow (cytometry) cohorts. The retrospective cohort consisted of 202 patients treated at the Cancer Center of Kuopio University Hospital, Finland, during 2015–2022. The flow cohort included 45 patients treated at the Cancer Centers of Oulu or Kuopio University Hospitals, Finland, between January 1st, 2020, and December 31st, 2023. Patients aged > 18 years with no prior ICI therapies and approved indications for ICI therapy were included. Dual ICI therapies (i.e. combinations of anti-PD(L)1 and anti CTLA4 antibodies) and ICIs combined with chemotherapy or TKIs were allowed. Age, sex, cancer type, metastatic burden and previous anti-cancer drugs used were documented as baseline demographic characteristic. The metastatic burden was assessed radiologically before ICI therapy initiation from the following sites: lymph nodes, brain, lung, liver, bone, and other.

### Ethics

The study was conducted under the research permits from Wellbeing Services county of Northern Savo and North Osthrobonia (diary no.s 59/13.00/2023 and 27/2020) and the acceptance of the Ethical Committees of Wellbeing Services counties of these regions (diary no.s 1482/2019 and 2/2020). For the prospective flow cohort, eligible patients were identified by their treating physicians at routine oncologist appointments and recruited by a member of the research group. All participants have given written informed consent, as mandated by the Declaration of Helsinki.

### Peripheral blood sample collection and storage

Complete blood counts, including the total leucocyte, neutrophil, monocyte and lymphocyte counts, were analyzed during routine lab blood sampling using a XN-1000 automated hematology analyzer (Sysmex). The results were retrospectively collected from electronic patient records. The blood tests were drawn 1–14 days before the first cycle of ICI treatment (pre-ICI) and 1–7 days before the second cycle of treatment (post-ICI). The blood count values are presented as x10E9/L.

In the prospective cohort, blood samples for flow cytometry were collected on average four days before (pre-ICI samples) and 24 days after (post-ICI samples) the initiation of ICI therapy. Following sample collection, peripheral blood mononuclear cells (PBMCs) were isolated from heparinized peripheral blood samples using Ficoll density gradient centrifugation with SepMate tubes (StemCell) and subsequently stored in liquid nitrogen.

### Flow cytometry

Altogether, 44 pre-ICI samples, and 41 post-ICI samples were analyzed. For analysis, PBMCs were thawed, treated with DNAse I (StemCell) according to manufacturer’s instructions, and aliquoted for surface marker staining. Six panels were used for stainings. The detailed staining protocol and panels are described in Supplemental Table 1. Representative gatings are presented in Supplemental Figs. 1, 2 and 3.

**Table 1:** Demographic characteristics of the retrospective and flow cohorts divided into therapy responders and non-responders

>	Retrospective cohort			Flow cohort		
Response status	All	Responder	Non-responder	All	Responder	Non-responder
n	202	86	115	45*	15	29
Age (yrs) ± SD	67.2 ± 10.7	67.9 ± 10.7	66.1 ± 10.7	67.5 ± 8.4	68.2 ± 8.0	66.9 ± 8.9
Gender						
Female	63 (31.2)	26 (30.2)	37 (32.2)	14 (31.1)	3 (20.0)	11 (37.9)
Male	139 (68.8)	60 (69.8)	78 (67.8)	31 (68.9)	12 (80.0)	18 (62.1)
Cancer type						
NSCLC^#^	96 (47.5)	48 (55.8)	48 (41.7)	25 (55.6)	12 (80.0)	12 (41.4)
Melanoma	37 (18.3)	10 (11.6)	27 (23.5)	12 (26.7)	2 (13.3)	10 (34.5)
RCC^##^	30 (14.9)	11 (12.8)	18 (15.7)	8 (17.8)	1 (6.7)	7 (24.1)
Other	39 (19.3)	17 (19.8)	22 (19.1)			
Performance status						
0	110 (54.5)	56 (65.1)	53 (46.1)	20 (44.4)	9 (60.0)	11 (37.9)
≥ 1	92 (45.5)	30 (34.9)	62 (53.9)	25 (55.6)	6 (40.0)	18 (62.1)
Metastatic burden & sites						
1–2 metastatic sites	140 (69.3)	75 (87.2)	64 (55.7)	26 (57.8)	12 (80.0)	13 (44.8)
> 2 metastatic sites	62 (30.7)	11 (12.8)	51 (44.3)	19 (42.2)	3 (20.0)	16 (55.2)
Lymph node	130 (64.4)	54 (62.8)	76 (66.1)	31 (68.9)	10 (66.7)	20 (69.0)
Brain	20 (9.9)	6 (7.0)	14 (12.2)	5 (11.1)	2 (13.3)	3 (10.3)
Lung	90 (44.6)	41 (47.7)	48 (41.7)	19 (42.2)	7 (46.7)	12 (41.4)
Liver	37 (18.3)	4 (4.7)	33 (28.7)	6 (13.3)	2 (13.3)	4 (13.8)
Bone	56 (27.7)	16 (18.6)	40 (34.8)	10 (22.2)	2 (13.3)	8 (27.6)
Treatment						
Nivolumab	55 (27.2)	19 (22.1)	35 (30.4)	13 (28.9)	2 (13.3)	11 (37.9)
Pembrolizumab	87 (43.1)	34 (39.5)	53 (46.1)	18 (40.0)	6 (40.0)	11 (37.9)
Atezolizumab	6 (3.0)	1 (1.2)	5 (4.3)	0 (0.0)	0 (0.0)	0 (0.0)
Nivolumab + Ipilimumab	10 (5.0)	5 (5.8)	5 (4.3)	7 (15.6)	3 (20.0)	4 (13.8)
ICI + chemotherapy	42 (20.8)	26 (30.2)	16 (13.9)	7 (15.6)	4 (26.7)	3 (10.3)
Nivolumab + TKI^###^	2 (1.0)	1 (1.2)	1 (0.9)	0 (0.0)	0 (0.0)	0 (0.0)
Treatment line						
1	122 (60.4)	58 (67.4)	64 (55.7)	35 (77.8)	12 (80.0)	23 (79.3)
≥ 2	80 (39.6)	28 (32.6)	51 (44.3)	10 (22.2)	3 (20.0)	6 (20.7)
Median follow-up time (months)	37.6 mo	41.0 mo	33.8 mo	28.9 mo	26.0 mo	N/A mo

^*^One responder was excluded from the cohort to lack of a pre-ICI blood sample

^#^NSCLC = non-small cell lung carcinoma

^#^^#^renal cell carcinoma

^###^ nivolumab + tyrosine kinase inhibitor

**Fig. 1:**
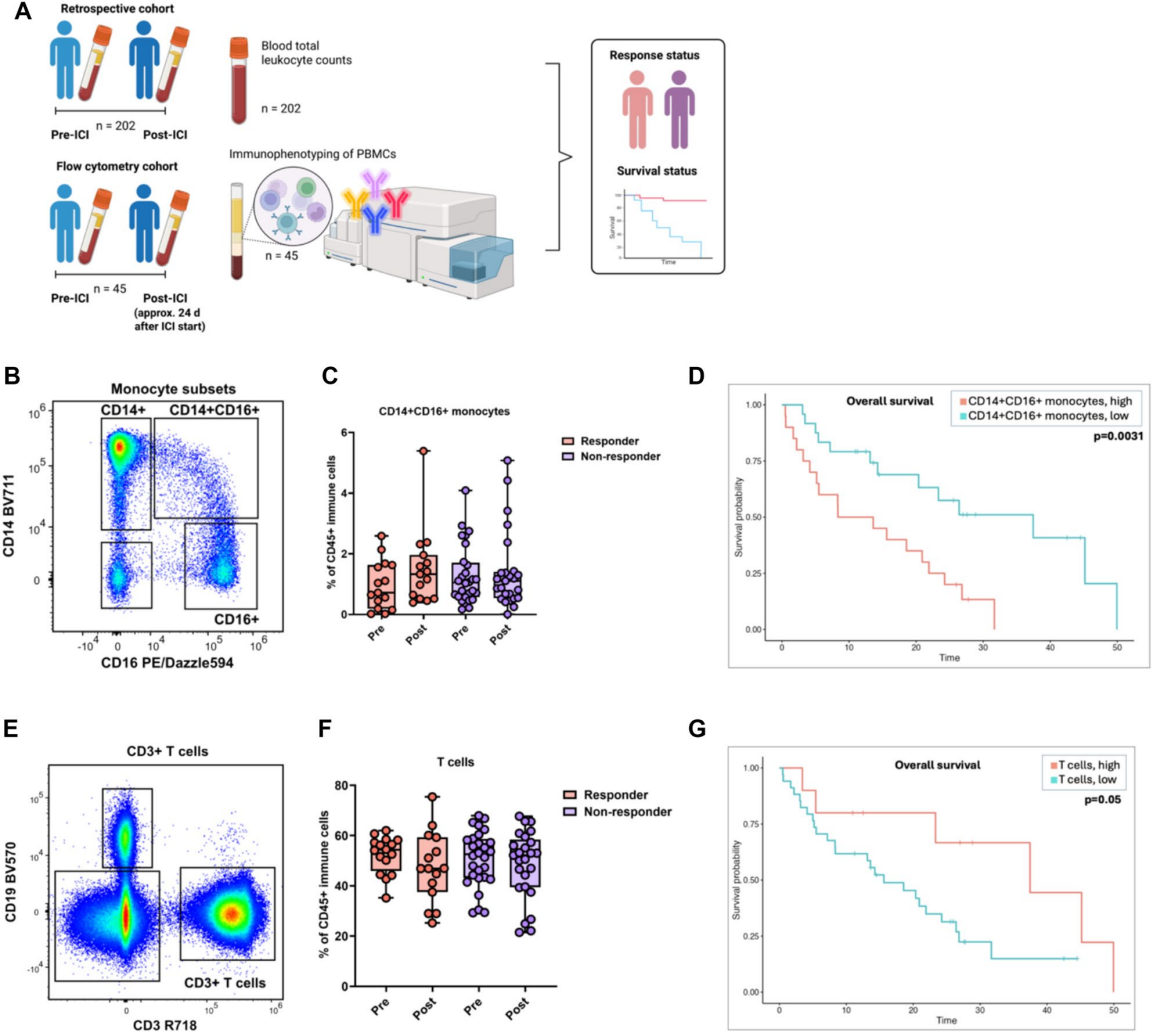
Experimental layout and demonstration of main findings. (**A**). Schematic representation of the experimental layout **(B)**. Representative gating of monocyte subtypes **(C)**. Frequencies of CD14^+^CD16^+^ intermediate monocytes in the pre- and post-treatment samples according to the response groups **(D)**. Overall survival of patients with high and low pre-treatment frequencies of CD14^+^CD16^+^ intermediate monocytes **(E)**. Representative gating strategy for CD3^+^ T cells **(F)**. Frequencies of T cells in pre- and post-treatment samples according to the response groups **(G)**. Overall survival of patients with high and low pre-treatment frequencies of T cells. ICI, immune checkpoint inhibitors; PBMC, peripheral blood monocytes

**Fig. 2:**
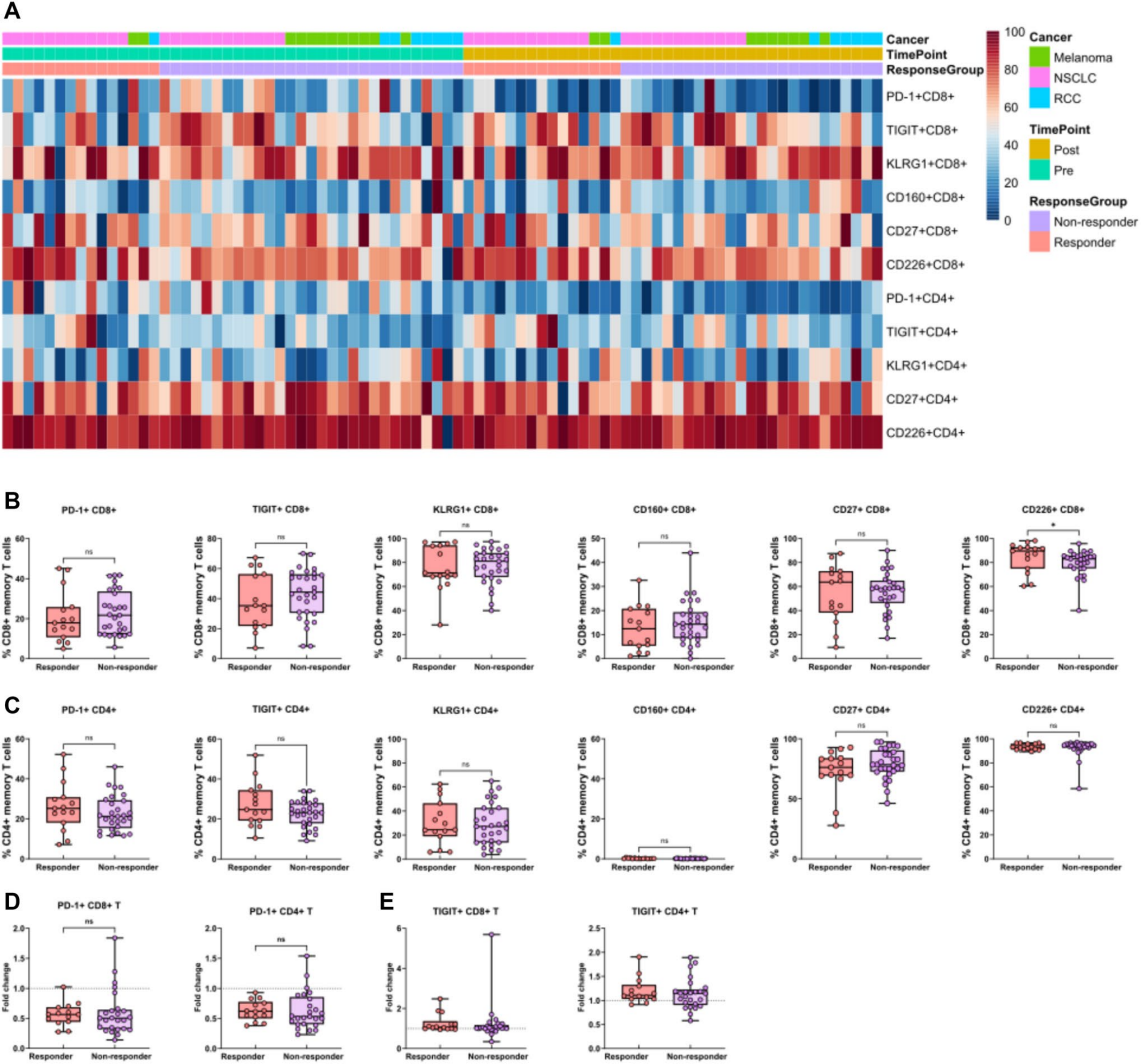
Co-inhibitory and co-activation receptor expressing circulating CD8^+^ and CD4^+^ T cells in patients receiving ICI therapy. **A**. Heatmap of CD8^+^ and CD4^+^ T cells expressing surface receptors PD-1, TIGIT, KLRG1, CD160, CD27 and CD226 in pre- and post-treatment samples. The cell frequencies were normalized for visualization **B**. Pre-treatment frequencies of PD-1, TIGIT, KLRG1, CD160, CD27 and CD226 in CD8^+^ and CD4^+^ memory T cells **C**. Fold change of **D** PD-1 and **E** TIGIT in memory CD8^+^ and CD4^+^ T cells in responders and non-responders. Mann–Whitney U was used for statistical testing in B-E. Fold change 1 (no change) is indicated as dotted line in D-E

**Fig. 3:**
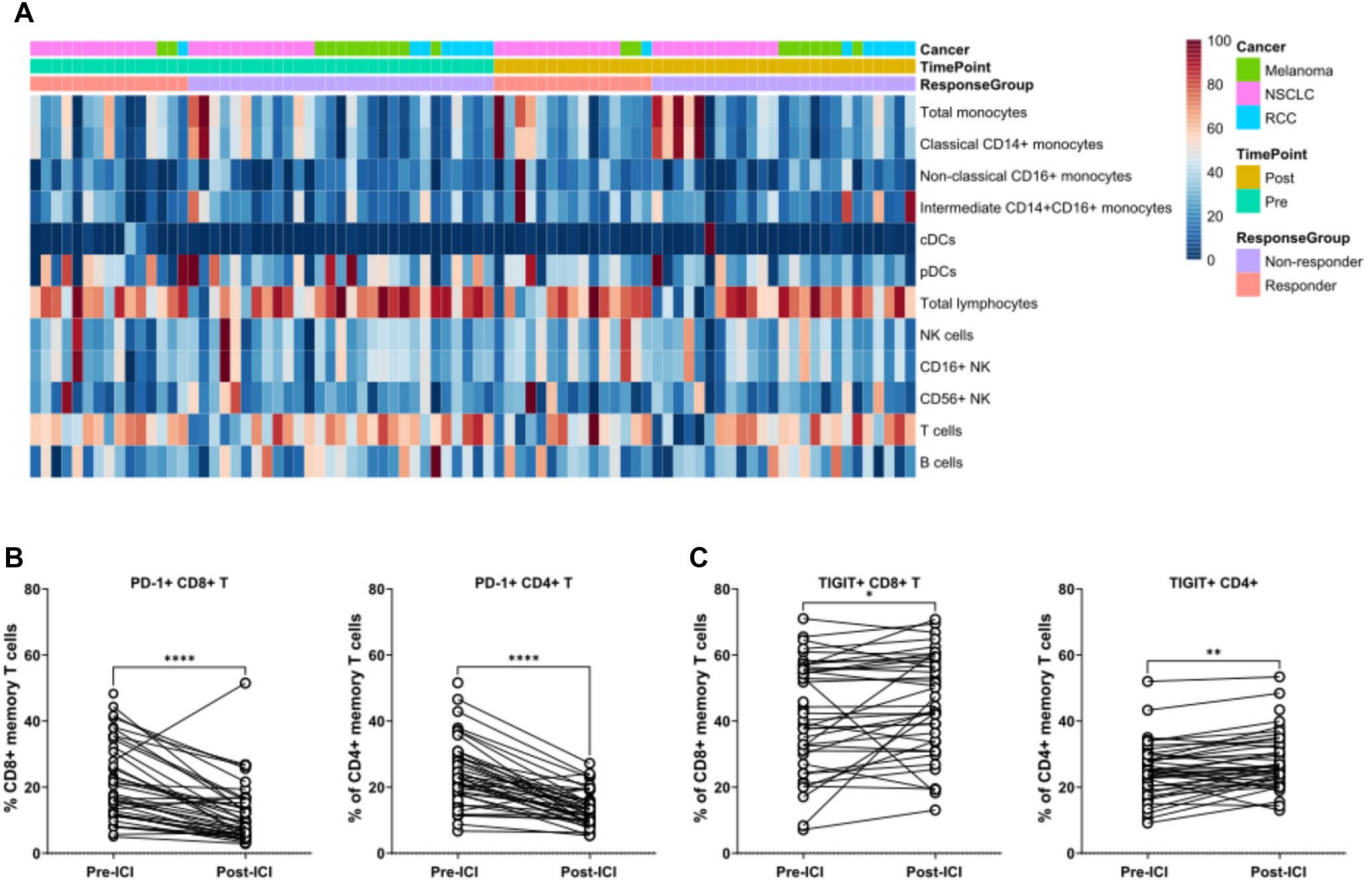
Heatmap of the immune cell landscape depicting immune cell frequencies pre and post ICI therapy. The cell frequencies were normalized for visualization. **A** Frequency of PD-1 **B** and TIGIT **C** expressing memory CD4^+^ and CD8^+^ T cells pre and post ICI treatment. Wilcoxon test was used for statistical testing in B-C. *P < 0.05, **P < 0.01, ****P < 0.0001. cDCs, classical dendritic cells; pDCs, Peripheral blood mononuclear cells; NK, natural kills

### Clinical outcome assessment

The clinical outcomes assessed were radiological response, progression free survival (PFS), and overall survival (OS). The radiological response was assessed from the first contrast-enhanced computed tomography (CT) scans conducted after ICI initiation (at 8–12 weeks) by a radiology specialist using the RECIST criteria. The responses were categorized either as complete response (CR), partial response (PR), stable disease (SD), or progressive disease (PD). For data analysis, CR and PR were grouped as responders (R), while SD and PD were categorized as non-responders (NR) (Supplemental Table (Suppl. Table) 2). Progression-free survival (PFS) was defined as the time from the date of ICI initiation to either cancer progression, death from any cause or the end of follow-up (FU), whichever occurred first. Overall survival (OS) was defined from the date of ICI treatment initiation to the date of death from any cause or the end of follow-up (FU), whichever occurred first. To assess the longitudinal changes in cell frequencies and phenotypes between pre- and post-therapy time points, post-treatment cell counts divided by pre-treatment cell counts were calculated (fold change).

### Statistical analyses

For the flow cytometry data, statistical analyses were conducted using GraphPad Prism (version 10, GraphPad Software Inc). The Wilcoxon rank sum test was used to compare pre- and post-ICI initiation samples, while Mann Whitney U test was applied for comparing responder and non-responder groups. Pairwise correlations between major blood immune cell subtypes were assessed using Spearman's rank correlation with Benjamini–Hochberg false discovery rate (FDR) correction [12]. The prospective cohort utilized for flow cytometry analyses was relatively small, and due to the exploratory nature of the analyses, correction for multiple comparisons was not performed. Survival analyses were performed in SPSS statistics software (version 27, IBM SPSS Statistics for Windows, IBM, Armonk, NY, USA) using the Kaplan–Meier method with Log Rank test. *P*-values < 0.05 were considered statistically significant. The patients were grouped into dichotomic PFS and OS subgroups based on immune cell cut offs determined by maximizing the log-rank test statistics in the two-group survival analysis in a Cox regression model, as previously demonstrated by Contal and O’Quigley [13]. The cut offs and the number of records and events in each subgroup are presented in the Supplemental Table 3. These cut offs were also used for NSCLC subgroup analyses (Suppl. Table 4). Cut offs based on subgroups of < 10 records were discarded. Benjamini–Hochberg false discovery rate adjusted p-values (FDR) are reported alongside uncorrected *p*-values, where indicated (Table 2, Suppl. Table 4, 7). The correlation heatmap was drawn with “pheatmap” R-package using in-house scripts in Rstudio [14–16].

**Table 2:** The association of pre-treatment counts/frequencies and fold changes of blood immune cells with radiological response, PFS and OS in the retrospective and flow* study cohorts divided into **A)** total leukocytes, neutrophils and the subpopulations of the monocytic lineage, and **B)** total lymphocytes, NK, T and B cells. Statistically significant results are bolded

(A) Endpoint			Radiological early response								PFS							**OS**						
Indicator			Pre-treatment median frequency/proportion (SD)				Median fold change* (SD)				HR (95% CI)			mPFS months				HR (95% CI)			mOS months			
Grouping criterion			R responder NR non-responder				R responder NR non-responder							> cell frequency/ proportion higher than cutoff < lower than cutoff							> cell frequency/ proportion higher than cutoff < lower than cutoff			
Group			R	NR	*p*	*FDR*	R	NR	*p*	*FDR*		*p*	*FDR*	>	≤	*p*	*FDR*		*p*	*FDR*	>	≤	*p*	*FDR*
Cell population	LEUCOCYTES	Leukocytes (E9/L)	7.05 (3.86)	7.00 (3.62)	0.78	0.96	0.99 (0.26)	0.96 (0.39)	0.56	0.99	1.03 (0.99–1.07)	0.18	0.48	**2.2** **(1.8–13.2)**	**6.5** **(5.3–8.5)**	**0.02**	**0.04**	**1.07** **(1.02–1.12)**	**0.003**	**0.02**	**14.4** **(10.3–20.9)**	**24.5** **(19.4–31.2)**	**0.006**	**0.01**
	NEUTROPHILS	Neutrophils (E9/L)	4.50 (3.78)	4.80 (2.81)	0.24	0.92	0.97 (0.44)	0.91 (0.45)	0.98	0.99	1.03 (0.98–1.07)	0.23	0.53	**4.8** **(3.5–7.4)**	**7.8** **(5.5–10.3)**	**0.02**	**0.04**	**1.06** **(1.01–1.11)**	**0.01**	**0.04**	**13.8** **(9.2–19.8)**	**26.9** **(20.0–31.7)**	**0.002**	**0.01**
	MONOCYTIC LINEAGE	Monocytes (E9/L)	0.50 (0.22)	0.50 (0.31)	0.60	0.92	1.00 (0.86)	1.00 (0.53)	0.09	0.44	**2.61** **(1.14–5.95)**	**0.02**	**0.16**	**3.2** **(0.6–5.7)**	**7.1** **(3.9–10.3)**	**0.02**	**0.04**	**5.09** **(1.98–13.07)**	**0.001**	**0.02**	**7.5** **(6.3–8.7)**	**22.7** **(17.1–28.4)**	**0.003**	**0.01**
		Monocytes*	22.75 (10.59)	21.36 (12.52)	0.98	0.98	1.08 (0.72)	1.08 (0.54)	0.94	0.99	1.01 (0.97–1.04)	0.74	0.91	N/A	N/A	N/A	N/A	1.03 (1.00–1.06)	0.08	0.25	**18.6** **(8.3–24.3)**	**37.5** **(14.3–N/A)**	**0.01**	**0.02**
		Classical monocytes*	19.6 (10.26)	19.6 (11.73)	0.95	0.98	1.01 (0.78)	1.05 (0.59)	0.92	0.99	1.00 (0.97–1.03)	0.95	0.95	7.4 (2.7–14.5)	2.2 (0.8–N/A)	0.16	0.18	1.03 (0.99–1.06)	0.12	0.25	**15.6** **(8.3–24.3)**	**45.2** **(14.3–N/A)**	**0.005**	**0.01**
		Non-classical monocytes*	0.53 (0.68)	0.93 (0.73)	0.42	0.92	1.15 (4.49)	0.97 (1.23)	0.11	0.44	1.55 (0.96–2.51)	0.07	0.28	**2.6** **(1.6–14.3)**	**12.2** **(2.8–N/A)**	**0.04**	**0.05**	1.33 (0.79–2.24)	0.28	0.45	14.9 (5.5–37.5)	24.3 (20.4–N/A)	0.22	0.23
		Intermediate monocytes*	0.72 (0.80)	1.03 (0.97)	0.31	0.92	1.72 (10.10)	1.13 (2.66)	0.07	0.44	**1.43** **(1.01–2.04)**	**0.04**	**0.21**	**2.7** **(1.9–13.2)**	**12.4** **(2.8–N/A)**	**0.03**	**0.05**	**1.60** **(1.10–2.31)**	**0.01**	**0.04**	**11.0** **(5.2–24.3)**	**37.5** **(20.4–N/A)**	**0.003**	**0.01**
		Dendritic cells cDCs*	0.51 (3.27)	0.47 (0.32)	0.75	0.96	0.97 (1.09)	1.15 (4.11)	0.53	0.99	0.80 (0.53–1.22)	0.31	0.62	N/A	N/A	N/A	N/A	0.88 (0.58–1.32)	0.54	0.72	N/A	N/A	N/A	N/A
		Dendritic cells pDCs*	0.25 (0.17)	0.18 (0.14)	0.58	0.92	1.10 (0.75)	0.76 (0.78)	0.08	0.44	0.89 (0.11–7.59)	0.92	0.95	N/A	N/A	N/A	N/A	0.94 (0.10–8.57)	0.96	0.96	N/A	N/A	N/A	N/A

(B)Endpoint			Radiological response								PFS							**OS**						
Indicator			Pre-treatment median frequency (SD)				Median fold change* (SD)				HR (95% CI)			mPFS months				HR (95% CI)			mOS months			
Grouping criterion			R responder NR non-responder				R responder NR non-responder							> cell frequency/ proportion higher than cutoff < lower than cutoff							> cell frequency/ proportion higher than cutoff < lower than cutoff			
Group			R	NR	*p*	*FDR*	R	NR	*p*	*FDR*		*p*	*FDR*	>	≤	*p*	*FDR*		*p*	*FDR*	>	≤	*p*	*FDR*
Cell population	LYMPHO-CYTES	Lymphocytes (E9/L)	1.70 (0.70)	1.60 (0.76)	0.51	0.92	1.00 (0.24)	1.00 (0.24)	0.80	0.99	0.82 (0.64–1.04)	0.09	0.29	**7.4** **(5.5–9.6)**	**4.5** **(2.9–6.5)**	**0.006**	**0.04**	0.80 (0.60–1.05)	0.11	0.25	**24.3** **(16.8–31.7)**	**18.6** **(13.5–23.6)**	**0.015**	**0.02**
		Lymphocytes*	71.5 (9.73)	73.9 (12.87)	0.52	0.92	1.04 (0.23)	0.96 (0.21)	0.25	0.80	1.0 (0.97–1.04)	0.84	0.91	N/A	N/A	N/A	N/A	0.98 (0.95–1.01)	0.14	0.25	37.5 (7.2–N/A)	19.5 (13.1–26.4)	0.07	0.09
	NK CELLS	Total NK cells*	11.60 (7.44)	12.70 (6.90)	0.63	0.92	1.05 (0.86)	1.09 (0.81)	0.44	0.99	1.01 (0.96–1.06)	0.71	0.91	N/A	N/A	N/A	N/A	0.99 (0.94–1.04)	0.66	0.75	N/A	N/A	N/A	N/A
		CD16 + NK cells*	11.10 (7.97)	12.3 (6.53)	0.61	0.92	1.02 (56.9)	1.09 (1.32)	0.41	0.99	1.01 (0.96–1.06)	0.60	0.87	2.8 (2.0–13.2)	7.7 (2.5–N/A)	0.37	0.37	0.99 (0.94–1.04)	0.71	0.76	20.9 (14.3–N/A)	13.1 (5.2–N/A)	0.23	0.23
		CD56 + NK cells*	0.14 (0.25)	0.24 (0.19)	0.40	092	0.91 (0.86)	0.85 (266.5)	0.99	0.99	1.64 (0.38–7.02)	0.50	0.80	N/A	N/A	N/A	N/A	1.96 (0.45–8.51)	0.37	0.54	N/A	N/A	N/A	N/A
	T CELLS*		54.1 (7.48)	53.1 (10.96)	0.91	0.98	0.93 (0.20)	0.97 (0.23)	0.65	0.99	0.99 (0.95–1.02)	0.45	0.80	N/A	N/A	N/A	N/A	0.97 (0.94–1.01)	0.13	0.25	**37.5** **(23.3–N/A)**	**15.6** **(8.3–26.4)**	**0.05**	**0.07**
	B CELLS*		3.68 (2.60)	4.40 (4.80)	0.41	0.92	0.88 (0.62)	0.87 (0.50)	0.76	0.99	**1.12** **(1.02–1.23)**	**0.02**	**0.16**	N/A	N/A	N/A	N/A	1.02 (0.93–1.13)	0.63	0.75	N/A	N/A	N/A	N/A

^*^Prospective flow cytometry cohort; FDR, FDR-adjusted p-value

## Results

### Patient characteristics

The baseline characteristics of patients in the retrospective (n = 202) and flow (n = 45) cohorts are shown in Table 1. The median ages were 67.2 years and 67.5 years, respectively. In both cohorts, there was a male dominance (68.8% and 68.9%) (Table 1).

Non-small cell lung cancer (NSCLC) and melanoma were the most common types of cancer in both cohorts, followed by renal cell carcinoma (RCC). 69.3% of the patients in the retrospective and 57.8% in the flow cohort had less than three metastatic sites involved, as assessed from CT scans before ICI therapy. The metastatic burden was higher in non-responders in comparison to responders in both retrospective (> 2 metastatic sites 44.3% vs. 12.8%, *p* < 0.05) and flow cohorts (55.2% vs. 20.0%, *p* < 0.05). The PS was higher in non-responders than in responders in the retrospective cohort (PS ≥ 1 53.9% vs. 34.9%, *p* < 0.05). Most patients in both cohorts received single ICIs as treatment. (Table 1) In the retrospective cohort, there were more responders in the ICI + chemotherapy/TKI group (responders 31.4% vs. 14.8%) than in the patients receiving single/doublet-ICI (responders 68.6% vs. 85.2%, *p* < 0.05).

### The association of leucocyte and neutrophil counts with clinical outcomes

We first analyzed the association of routine pre-treatment leukocyte subset counts with ICI therapy response and survival in the retrospective cohort. Total pre-treatment leukocyte and neutrophil counts between the responder and non-responder groups were comparable, and there were no significant alterations from pre- to post-treatment counts (fold change). However, higher pre-treatment leukocyte counts were significantly associated with poorer OS (Hazard ratio (HR) 1.07, 95% Confidence interval (CI) 1.02–1.12, *p* = 0.003, FDR = 0.02; mOS 14.4 vs. 24.5 mo., *p* = 0.006, FDR = 0.01). Similarly, higher pre-treatment neutrophil counts tended to associate with shorter PFS (HR 1.03, 95% CI 0.98–1.07, *p* = 0.23, FDR = 0.53; mPFS 4.8 vs. 7.8 mo., *p* = 0.02, FDR = 0.04) and OS (HR 1.06, 95% CI 1.01–1.11, *p* = 0.01, FDR = 0.04; mOS 13.8 vs. 26.9 mo., *p* = 0.002, FDR = 0.01) (Table 2A, Suppl. Figure 4). Of note, we conducted these analyses separately in patients with NSCLC histology (Suppl. Table 4), the largest cancer subgroup, and the findings were largely similar to the main tumor-agnostic cohort.

### The association of monocyte counts and their subtype frequencies with clinical outcomes

In the retrospective cohort, the pre-treatment monocyte counts, and the fold changes were similar between the response groups. Higher pre-treatment monocyte counts were associated with shorter PFS (HR 2.61, 95% CI 1.14–5.95, *p* = 0.02, FDR = 0.16; mPFS 3.2 vs. 7.1 mo., *p* = 0.021, FDR = 0.04) and OS (HR 5.09, 95% CI 1.98–13.07, *p* = 0.001, FDR = 0.02; mOS 7.5 vs. 22.7 mo., *p* = 0.003, FDR = 0.01) (Table 2A).

In the more detailed characterization of the monocytic cell lineage, no major differences were observed in the pre-treatment frequencies of the classical CD14^+^, non-classical CD16^+^ and intermediate CD14^+^CD16^+^ monocytes (Fig. 1C) or the DC subsets (plasmacytoid and classical DCs) between response groups, or when comparing fold changes (Table 2A). Overall, the lower frequency of all three monocyte subsets studied tended to associate with better survival (Table 2A). The strongest association was seen with intermediate (CD14^+^CD16^+^) monocytes (PFS: HR 1.43, 95% CI 1.01–2.04, *p* = 0.04, FDR = 0.21, mPFS 2.7 vs. 12.4 mo., *p* = 0.03, FDR = 0.05; OS HR 1.60, 95% CI 1.10–2.31, *p* = 0.01, FDR = 0.04; mOS 11.0 vs. 37.5 mo., *p* = 0.003, FDR = 0.01) (Table 2A, Fig. 1D). Significant associations between DC subsets and response or survival were not seen.

### The association of total lymphocyte counts and their subset frequencies with clinical outcomes

The pre-treatment lymphocyte count did not associate with radiological response but tended to be associated with better PFS (HR 0.82, 95% CI 0.64–1.04, *p* = 0.09, FDR = 0.29; mPFS 7.4 vs. 4.5 mo., *p* = 0.006, FDR = 0.04) and OS (HR 0.80, 95% CI 0.60–1.05, *p* = 0.11, FDR = 0.25; mOS 24.3 vs. 18.6 mo., *p* = 0.002, FDR = 0.02) (Table 2B, Suppl. Figure 4). Similar tendency for OS was observed in the flow cohort (HR 0.98, 95% CI 0.95–1.01, *p* = 0.14, FDR = 0.25; mOS 37.5 vs. 19.5 mo., *p* = 0.07, FDR = 0.09).

In the flow analyses, the pre-treatment frequencies of total NK, T, and B cells between the responder groups were comparable (Table 2B), as well as the frequencies of the CD16^+^ and CD56^+^ NK cell subsets, main naïve and memory T cell subsets (defined by CD45RA/CCR7/CD27 expression), or T regulatory cells (CD4^+^CD127^low^CD25^+^), and main naïve and memory B cells (defined by CD27/IgD expression) (Suppl. Table 5). Small, albeit significant, alterations were observed as a slightly higher frequency of CD27^−^IgD^−^ double-negative (DN) memory B cells (7.0 vs. 4.8, *p* = 0.0497 Suppl. Table 5) in treatment responders and a fold decrease of CD19^+^ unswitched memory (CD27^+^IgD^+^) B cells (1.067 vs. 0.8986, p = 0.0279); Suppl. Table 5) in non-responders. In survival analyses, a higher pre-treatment T cell frequency tended to be associated with better OS (HR 0.97, 95% CI 0.94–1.01, *p* = 0.13, FDR = 0.25; mOS 37.5 vs 15.6 mo., *p* = 0.05, FDR = 0.07) (Table 2B, Fig. 1G). Moreover, the frequency of T cells correlated negatively with the frequency of intermediate (r = − 0.53, *p* < 0.005) and non-classical (r = − 0.64, *p* < 0.0005) monocytes (Suppl. Figure 5). In Cox analysis, a higher pre-treatment B cell frequency was associated with shorter PFS (HR 1.12, 95% CI 1.02—1.23, *p* = 0.02, FDR = 0.16). Significant associations between NK cell populations or their subtypes with survival were not observed.

### The uni- and multivariate Cox regression analysis of main immune cell classes and clinical parameters

Alongside laboratory parameters, age, gender, WHO PS, metastatic burden, and treatment line were selected as clinical parameters for Cox regression analyses (Table 3). In univariate analyses, significant associations between leucocytes, neutrophils and monocytes and survival were seen, as previously presented in Table 2A. PS was associated with poorer PFS (HR 1.80, 95% CI 1.31–2.46, *p* < 0.001) and OS (HR 2.28, 95% CI 1.60–3.25, *p* < 0.001), along with metastatic burden (PFS HR 2.61, 95% CI 1.88–3.63, *p* < 0.001; OS HR 2.38, 95% CI 1.67–3.39, *p* < 0.001, Table 3A). Significant associations in the univariate analysis were chosen for multivariate analysis. In multivariate analysis (Table 3B), the clinical variables WHO PS (PFS: HR 2.25, 95% CI 1.39–3.65, *p* < 0.001; OS: HR 1.97, 95% CI 1.17–3.30, *p* = 0.010) and metastatic burden (PFS: HR 2.20, 95% CI 1.35–3.58, *p* = 0.002; OS: HR 1.77, 95% CI 1.07–2.94, *p* = 0.027) were significantly associated with survival. Out of blood cell counts, only monocytes retained significance for OS (HR 4.56, 95% CI 1.07–19.51, *p* = 0.041).

**Table 3:** (A) Uni- and (B) multivariate Cox regression analysis in the retrospective cohort including clinical and laboratory pre-treatment variables. Statistically significant results are bolded

A)Univariate				
	PFS		OS	
	HR (95% Cl)	*p*	HR (95% Cl)	*p*
Gender (0 female, 1 male)	0.82 (0.58–1.15)	0.244	0.77 (0.53–1.13)	0.178
Age	0.99 (0.98–1.00)	0.252	0.99 (0.97–1.00)	0.097
PS (WHO 0 vs. ≥ 1)	**1.80 (1.31–2.46)**	** < 0.001**	**2.28 (1.60–3.25)**	** < 0.001**
Metastatic burden (1–2 vs. ≥ 3)	**2.61 (1.88–3.63)**	** < 0.001**	**2.38 (1.67–3.39)**	** < 0.001**
Treatment line (0 vs. ≥ 1)	1.28 (0.94–1.75)	0.121	1.34 (0.95–1.89)	0.096
Leukocytes (E9/L)	1.03 (0.99–1.07)	0.182	**1.07 (1.02–1.12)**	**0.003**
Neutrophils (E9/L)	1.03 (0.98–1.07)	0.225	**1.06 (1.01–1.11)**	**0.011**
Monocytes (E9/L)	**2.61 (1.14–5.96)**	**0.023**	**5.09 (1.98–13.07)**	** < 0.001**
Lymphocytes (E9/L)	0.82 (0.64–1.04)	0.099	0.80 (0.60–1.05)	0.110

B) Multivariate				
	PFS		OS	
	HR (95% Cl)	*p*	HR (95% Cl)	*p*
PS (WHO 0 vs. ≥ 1)	**2.25 (1.39–3.65)**	** < 0.001**	**1.97 (1.17–3.30)**	**0.010**
Metastatic burden (1–2 vs. ≥ 3)	**2.20 (1.35–3.58)**	**0.002**	**1.77 (1.07–2.94)**	**0.027**
Leukocytes (E9/L)	0.92 (0.68–1.24)	0.578	0.58 (0.58–1.10)	0.174
Neutrophils (E9/L)	1.06 (0.81–1.39)	0.694	1.29 (0.97–1.73)	0.082
Monocytes (E9/L)	3.23 (0.85–1.24)	0.084	**4.56 (1.07–19.51)**	**0.041**

### The association of the expression of co-inhibitory and co-activation receptors by T cells with clinical outcomes

As anti-PD(L)1 immunotherapy may lead to the upregulation of other co-inhibitory receptors as a mechanism of ICI resistance [17, 18], we next used flow cytometry to analyze the expression of co-inhibitory/co-activation receptors PD-1, TIGIT, KLRG1 (Killer cell lectin-like receptor G1), CD160, CD27, CD226, Tim-3 and LAG-3 on CD8^+^ and CD4^+^ memory T cells at baseline in treatment responders and non-responders (Fig. 2A). Tim-3 was minimally expressed by circulating T cells, as expected [19], as well as LAG-3 and CD160 by CD4^+^ memory T cells, and therefore these markers were not analyzed further (Suppl. Table 6).

A higher frequency of CD8^+^ memory T cells expressing the CD226 co-activation marker was observed in treatment responders (89.6% vs. 83.0%, *p* = 0.04; Fig. 2B). Otherwise, comparable pre-treatment frequencies and fold changes of co-inhibitory receptor-expressing CD8^+^ and CD4^+^ memory T cells were seen between the responder groups (Fig. 2A-E).

In survival analyses, a higher pre-treatment frequency of cells expressing the co-activation molecule CD226^+^ tended to be associated with better survival in both CD4^+^ (mPFS 16.6 vs. 2.7 mo., *p* = 0.05, FDR = 0.13; mOS 26.8 mo. vs. 13.6 mo., *p* = 0.06, FDR = 0.14) and CD8^+^ memory T cell compartments (mPFS 12.8 vs. 8.7 mo., *p* = 0.11, FDR = 0.14, mOS 26.8 vs. 13.1 mo., *p* = 0.02, FDR = 0.14) (Suppl. Table 7, Suppl. Figure 6).

### The overall effect of ICI therapy on circulating immune cells

Finally, to examine the overall pharmacological effect of ICI treatment on the immune cell landscape, we studied major immune cell frequencies regardless of the response status between pre- and post-treatment samples. No major differences were observed in the monocyte, DC or lymphocyte frequencies between pre- and post-treatment samples indicating that the immune cell subsets did not significantly deviate from baseline at the post-ICI timepoint (Fig. 3A, Suppl. Table 6). Only small, albeit significant, alterations within the lymphocyte compartment were observed, with a lower frequency of naïve CD8^+^ (*p* = 0.002) and total CD4^+^ T cells (*p* = 0.03), and a higher frequency of CD27^−^ TEMRA CD8^+^ T cells (*p* = 0.005) and CD27^+^ EM CD4^+^ T cells (*p* = 0.02) in the post-treatment compared to pre-treatment samples, regardless of the response groups (Suppl. Table 6).

When we studied the expression of PD-1, TIGIT, KLRG1, CD160, CD27, and CD226 in CD8^+^ and CD4^+^ memory T cells between the pre- and post-treatment samples, PD-1 expression was, as expected, significantly reduced in response to anti PD-(L)1 therapy in both CD8^+^ and CD4^+^ T cells (both p < 0.0001) (Fig. 3B, Suppl. Table 6). Small but significant increases in TIGIT^+^ CD8^+^ (*p* = 0.015) and TIGIT^+^ CD4^+^ memory T cells (*p* = 0.002) were also observed between the pre- and post-treatment samples (Fig. 3C, Suppl. Table 6).

## Discussion

In this study, we assessed the potential of routinely measured major blood immune cell counts and their subtypes to predict the response and survival of patients with advanced cancer undergoing ICI therapy. Higher total pre-treatment monocyte counts, and lower lymphocyte counts were associated with shorter survival. Using more detailed immune cell subtyping, decreased survival was associated particularly with higher intermediate monocyte and lower T cells frequencies. Notably, strong associations between major immune cell populations, or their extensively studied subsets including monocyte, NK, and B/T lymphocyte subtypes, and early radiological ICI response remained unobserved. Thus, rather than predicting early responders to ICIs, blood immune cell characterization may serve as a tool for stratifying long term survivors after ICI therapy.

A relative increase in myeloid cells with a concomitant decrease in lymphoid cells in peripheral blood has been consistently associated with poorer survival of patients with cancer in general [20–22], and reduced benefit from ICI therapy across tumor types [3, 4]. Our findings from the retrospective cohort were concordant with these studies and were further confirmed in an independent prospective flow cohort, in which we also observed a strong inverse correlation between monocyte and lymphocyte counts. Biologically, the observations most likely reflect the systemic inflammation related to the overall burden of the malignant disease [23] and are therefore predominantly prognostic in nature rather than predictive of ICI therapy. Further supporting this interpretation, the clinical variables reflecting disease burden retained independent prognostic significance in our multivariable Cox regression analysis, while the associations of blood counts with survival were largely attenuated.

Our flow subtyping of monocytes suggested that poorer prognosis was specifically associated with higher pretreatment frequencies of intermediate (CD14^+^CD16^+^) monocytes. Intermediate monocytes exhibit immunosuppressive properties functionally distinct from classical or non-classical monocytes [24] and appear to harbor M2 pro-tumorigenic phenotype with the capacity to infiltrate tumor-areas in patients with cancer [25]. Our current findings are consistent with a previous study by de Lima et al. [9] and support the concept that increased intermediate monocytes are linked with impaired T cell function which may attenuate ICI efficacy [9, 26]. However, results contrasting our study have also been reported [8, 27], and some studies have failed to identify any associations between monocytes and survival outcomes [6]. The discrepancies may be explained by the plasticity of monocytes, resulting in functional heterogeneity already at baseline and further phenotypic shifts during treatment [28]. Additionally, differences in cohort composition, heterogenicity in tumor histologies, methodological approaches, and sampling schedules are likely to have contributed to the inconsistent findings. In the future, larger prospective studies with well-defined cohorts will be needed to clarify the heterogeneity observed.

The characterization of lymphocyte subsets in our study revealed a trend towards longer OS in patients with elevated baseline T cell frequencies. These results are in line with prior studies where lower lymphocyte counts have been associated with progression [29] and a robust T-cell pool with prolonged survival [30]. We did not see clear associations between pre-treatment B cell and NK cell subset frequencies and OS nor signals of early increase in lymphocyte counts during ICI therapy—findings that contrast some of the previous studies [5, 31]. We may have missed very early, possibly transient immune cell dynamics due to a later collection of post-treatment samples (approx. 24 days after ICI initiation), and we also lacked cell proliferation markers from our flow staining panel.

Since resistance to ICI can be conveyed by not only PD-1 blockade but also other coinhibitory receptors, we wanted to investigate how PD-1 blockade affects the expression of other co-stimulatory/inhibitory receptors on circulating memory CD8^+^ and CD4^+^ T cells. When comparing pooled pre- and post-treatment samples, we found a decrease in the frequency of PD-1 expressing CD8^+^ and CD4^+^ memory T cells after therapy initiation, consistent with effective PD-1 receptor blockade in vivo. We also found an increased frequency of CD4^+^ and CD8^+^ memory T cells expressing TIGIT in the post-treatment samples. However, since these expression patterns were similar between responders and non-responders, they most likely represent general pharmacodynamic effects of PD-1 blockade rather than associate with biological efficacy of ICIs. The expression of other co-inhibitory receptors examined showed also minimal discriminatory capacity between responder groups. We observed, however, a slightly higher baseline frequency of CD8^+^ memory T cells expressing the co-activation marker CD226 in ICI responders, and trending signals towards longer survival in patients with higher baseline frequencies of both CD4^+^ and CD8^+^ memory T cells expressing CD226. Downregulation of CD226 on CD8^+^ T cells in tumors has previously been described as a potential mechanism of immune escape [34, 35]. Conversely, the increased expression of CD226 on CD8^+^ T cells appears to induce tumor destruction and predict response during PD-1 blockade [36], indicating the potential of CD226 expression as a predictive biomarker for ICI therapy. Our current results are in line with these papers but need further validation in a larger cohort.

Overall, our extensive immune profiling did not identify strong cell populations discriminating early radiological responders from non-responders. This may partly stem from the limitations of RECIST-based response criteria in immunotherapy, where radiological response does not always translate into meaningful survival benefit. This has been stated also in previous studies [32, 33]. ICI-associated phenomena such as pseudo-progression and delayed treatment responses can confound early radiological assessments of therapeutic efficacy, particularly when compared with the more rapidly manifesting effects of conventional chemotherapy. Indeed, in our study, patients exhibiting early radiological responses were more frequently treated with chemo-immunotherapy combinations rather than ICI monotherapy. Furthermore, upon subsequent CT imaging, 13% (6/45) of early non-responders in the flow cohort converted to responders (data not shown). Collectively, these findings suggest that early radiological responses may disproportionately reflect the chemotherapy-driven effects, while delayed immunotherapy responses may be misclassified as disease progression at early time points.

The strengths of the current study include the tumor-agnostic approach, combined use of a large retrospective cohort, and a separate, prospectively collected cohort that was more comprehensively immunophenotyped. Sample processing and flow cytometry analyses were conducted using standardized protocols while minimizing technical variability. The limits of this study include the relatively small flow cohort size, heterogeneity in tumor types and therapy regimens, which all limit the statistical power and complicate the interpretation of immune-biomarker associations. Importantly, while tumor heterogeneity increases generalizability, it also introduces significant heterogeneity that may confound biomarker associations. Additionally, the identified associations in the flow cohort are preliminary and require validation in larger, independent cohorts, specifically powered for these endpoints. Moreover, noise from systemic conditions unrelated to cancer may have failed to capture potentially relevant immune cell signals in the analyses.

In conclusion, our findings indicate that elevated pre-treatment levels of blood monocytes combined with reduced lymphocyte levels are tumor-agnostically associated with inferior survival outcomes in patients treated with ICIs. Specific subpopulations, such as intermediate monocytes and CD226⁺CD8⁺ memory T cells, may harbor potential as prognostic/predictive indicators and warrant further investigation. While detailed immunophenotyping of blood immune cell populations yields important biological insights into immune cell dynamics during ICI therapy, it provided limited incremental prognostic value beyond readily available major immune cell frequencies derived from routine blood counts.

## Supplementary Information

Below is the link to the electronic supplementary material.

Supplementary file1 (PDF 2548 KB)

## Data Availability

No datasets were generated or analysed during the current study.

## Abbreviations

cDCs: Classical dendritic cells
CR: Complete response
CT: Contrast-enhanced computed tomography
DC: Dendritic cell
FDR: False discovery rate
FU: Follow-up
HR: Hazard ratio
ICI: Immune checkpoint inhibitor
KLRG1: Killer cell lectin-like receptor G1
LAG-3: Lymphocyte-activation gene 3
NK: Natural killer
NR: Non-responder
NSCLC: Non-small cell lung cancer
OS: Overall survival
PBMC: Peripheral blood mononuclear cells
PD: Progressive disease
pDCs: Plasmacytoid dendritic cells
PFS: Progression free survival
PR: Partial response
PS: Performance status
R: Responder
RCC: Renal cell carcinoma
SD: Stable disease
TIGIT: T cell Ig and ITIM domain
TMB: Tumor mutational burden

## References

[1] Robert C (2020) A decade of immune-checkpoint inhibitors in cancer therapy. Nat Commun 11(1):3801–3803. 10.1038/s41467-020-17670-y 32732879 10.1038/s41467-020-17670-y PMC7393098

[2] Gong J, Chehrazi-Raffle A, Reddi S, Salgia R (2018) Development of PD-1 and PD-L1 inhibitors as a form of cancer immunotherapy: a comprehensive review of registration trials and future considerations. J Immunother Cancer 6(1):8–18. 10.1186/s40425-018-0316-z 29357948 10.1186/s40425-018-0316-z PMC5778665

[3] Huemer F, Lang D, Westphal T, Gampenrieder SP, Hutarew G, Weiss L et al (2019) Baseline Absolute Lymphocyte Count and ECOG Performance Score are associated with survival in advanced Non-Small Cell Lung Cancer undergoing PD-1/PD-L1 blockade. J Clin Med 8(7):1014. 10.3390/jcm8071014 31295966 10.3390/jcm8071014 PMC6678702

[4] Goldschmidt JH, Chou LN, Chan PK, Chen L, Robert N, Kinsey J et al (2023) Real-world outcomes of 18,186 metastatic solid tumor outpatients: baseline blood cell counts correlate with survival after immune checkpoint inhibitor therapy. Cancer Med 12(22):20783–20797. 10.1002/cam4.6645 37962239 10.1002/cam4.6645 PMC10709745

[5] Kamphorst AO, Pillai RN, Yang S, Nasti TH, Akondy RS, Wieland A et al (2017) Proliferation of PD-1+ CD8 T cells in peripheral blood after PD-1-targeted therapy in lung cancer patients. Proc Natl Acad Sci U S A 114(19):4993–4998. 10.1073/pnas.1705327114 28446615 10.1073/pnas.1705327114 PMC5441721

[6] Edner NM, Ntavli E, Petersone L, Wang CJ, Fabri A, Kogimtzis A et al (2023) Stratification of PD-1 blockade response in melanoma using pre-and post-treatment immunophenotyping of peripheral blood. Immunother Adv 3:1–13. 10.1093/immadv/ltad001 10.1093/immadv/ltad001 PMC9929715 36818683

[7] Olingy C, Alimadadi A, Araujo DJ, Barry D, Gutierrez NA, Werbin MH et al (2022) CD33 expression on peripheral blood monocytes predicts efficacy of anti-PD-1 immunotherapy against Non-Small Cell Lung Cancer. Front Immunol 13:842653. 10.3389/fimmu.2022.842653 35493454 10.3389/fimmu.2022.842653 PMC9046782

[8] Krieg C, Nowicka M, Guglietta S, Schindler S, Hartmann FJ, Weber LM et al (2018) High-dimensional single-cell analysis predicts response to anti-PD-1 immunotherapy. Nat Med 24(2):144–153 29309059 10.1038/nm.4466

[9] de Lima ABV, Hansen M, Spanggaard I, Rohrberg K, Reker Hadrup S, Lassen U et al (2021) Immune cell profiling of peripheral blood as signature for response during checkpoint inhibition across cancer types. Front Oncol 11:558248. 10.3389/fonc.2021.558248 33842304 10.3389/fonc.2021.558248 PMC8027233

[10] Tawbi HA, Schadendorf D, Lipson EJ, Ascierto PA, Matamala L, Castillo Gutiérrez E et al (2022) Relatlimab and Nivolumab versus Nivolumab in untreated advanced melanoma. N Engl J Med 386(1):24–34. 10.1056/NEJMoa2109970 34986285 10.1056/NEJMoa2109970 PMC9844513

[11] Chauvin JM, Pagliano O, Fourcade J, Sun Z, Wang H, Sander C et al (2015) TIGIT and PD-1 impair tumor antigen–specific CD8+ T cells in melanoma patients. J Clin Invest 125(5):2046–2058 25866972 10.1172/JCI80445 PMC4463210

[12] Benjaminit Y, Hochberg Y (1995) Controlling the False Discovery Rate: a practical and powerful approach to multiple testing. J R Stat Soc Series B Stat Methodol 57(1):289–300. 10.1111/j.2517-6161.1995.tb02031.x

[13] Mandrekar JN, Mandrekar SJ, Cha S. Cutpoint Determination Methods in Survival Analysis using SAS ®. 2003 Jan;

[14] Kolde R. pheatmap: Pretty Heatmaps. R package version 1.0.13. https://CRAN.R-project.org/package=pheatmap. 2019.

[15] R Core Team (2024) R: A language and environment for statistical computing. R Foundation for Statistical Computing, Vienna, Austria

[16] Posit team (2025) RStudio: Integrated development environment for R. Posit Software, PBC, Boston, MA

[17] Gettinger S, Choi J, Hastings K, Truini A, Datar I, Sowell R et al (2017) Impaired HLA class I antigen processing and presentation as a mechanism of acquired resistance to immune checkpoint inhibitors in lung cancer. Cancer Discov 7(12):1420–1435. 10.1158/2159-8290.CD-17-0593 29025772 10.1158/2159-8290.CD-17-0593 PMC5718941

[18] Limagne E, Richard C, Thibaudin M, Fumet JD, Truntzer C, Lagrange A et al (2019) Tim-3/galectin-9 pathway and mMDSC control primary and secondary resistances to PD-1 blockade in lung cancer patients. Oncoimmunology 8(4):e1564505. 10.1080/2162402X.2018.1564505 30906658 10.1080/2162402X.2018.1564505 PMC6422400

[19] Gao X, Zhu Y, Li G, Huang H, Zhang G, Wang F et al (2012) TIM-3 expression characterizes regulatory T cells in tumor tissues and is associated with lung cancer progression. PLoS ONE 7(2):e30676. 10.1371/journal.pone.0030676 22363469 10.1371/journal.pone.0030676 PMC3281852

[20] Li GP, Zhang D, Li MH, Yuan FF, Hou XJ, He DJ et al (2024) Association between the neutrophil-to-lymphocyte ratio and cancer in adults from NHANES 2005–2018: a cross-sectional study. Sci Rep 14(1):23678–23710. 10.1038/s41598-024-75252-0 39390050 10.1038/s41598-024-75252-0 PMC11467198

[21] Cupp MA, Cariolou M, Tzoulaki I, Aune D, Evangelou E, Berlanga-Taylor AJ (2020) Neutrophil to lymphocyte ratio and cancer prognosis: an umbrella review of systematic reviews and meta-analyses of observational studies. BMC Med 18(1):360. 10.1186/s12916-020-01817-1 33213430 10.1186/s12916-020-01817-1 PMC7678319

[22] Gu L, Li H, Chen L, Ma X, Li X, Gao Y et al (2016) Prognostic role of lymphocyte to monocyte ratio for patients with cancer: evidence from a systematic review and meta-analysis. Oncotarget 7(22):31926–31942. 10.18632/oncotarget.7876 26942464 10.18632/oncotarget.7876 PMC5077986

[23] Varner JA, Schmid MC (2010) Myeloid cells in the tumor microenvironment: modulation of tumor angiogenesis and tumor inflammation. J Oncol 2010:201026. 10.1155/2010/201026 20490273 10.1155/2010/201026 PMC2871549

[24] Skrzeczyńska-Moncznik J, Bzowska M, Loseke S, Grage-Griebenow E, Zembala M, Pryjma J (2008) Peripheral blood CD14high CD16+ monocytes are main producers of IL-10. Scand J Immunol 67(2):152–159 18201370 10.1111/j.1365-3083.2007.02051.x

[25] Subimerb C, Pinlaor S, Lulitanond V, Khuntikeo N, Okada S, McGrath MS et al (2010) Circulating CD14(+) CD16(+) monocyte levels predict tissue invasive character of cholangiocarcinoma. Clin Exp Immunol 161(3):471–479. 10.1111/j.1365-2249.2010.04200.x 20636398 10.1111/j.1365-2249.2010.04200.x PMC2962964

[26] Prat M, Le Naour A, Coulson K, Lemée F, Leray H, Jacquemin G et al (2020) Circulating CD14 high CD16 low intermediate blood monocytes as a biomarker of ascites immune status and ovarian cancer progression. J Immunother Cancer 8:e000472. 10.1136/jitc-2019-000472 32503947 10.1136/jitc-2019-000472 PMC7279676

[27] Rochigneux P, Lisberg A, Garcia A, Granjeaud S, Madroszyk A, Fattori S et al (2022) Mass cytometry reveals classical monocytes, NK Cells, and ICOS+ CD4+ T Cells associated with Pembrolizumab efficacy in patients with lung cancer. Clin Cancer Res 28(23):5136–5148. 10.1158/1078-0432.CCR-22-1386 36166003 10.1158/1078-0432.CCR-22-1386 PMC10085054

[28] Chavan R, Salvador D, Gustafson MP, Dietz AB, Nevala W, Markovic SN (2014) Untreated stage IV melanoma patients exhibit abnormal monocyte phenotypes and decreased functional capacity. Cancer Immunol Res 2(3):241–248. 10.1158/2326-6066.CIR-13-0094 24778320 10.1158/2326-6066.CIR-13-0094 PMC4007317

[29] Pan M, Alavi M, Herrinton LJ (2018) Association of inflammatory markers with disease progression in patients with metastatic melanoma treated with immune checkpoint inhibitors. Perm J 22(2):17. 10.7812/TPP/17-149 29616914 10.7812/TPP/17-149 PMC5882187

[30] Mazzaschi G, Facchinetti F, Missale G, Canetti D, Madeddu D, Zecca A et al (2019) The circulating pool of functionally competent NK and CD8+ cells predicts the outcome of anti-PD1 treatment in advanced NSCLC. Lung Cancer 127:153–163. 10.1016/j.lungcan.2018.11.038 30642544 10.1016/j.lungcan.2018.11.038

[31] Kim KH, Cho J, Ku BM, Koh J, Sun JM, Lee SH et al (2019) The first-week proliferative response of peripheral blood PD-1+CD8+ T Cells predicts the response to anti-PD-1 therapy in solid tumors. Clin Cancer Res 25(7):2144–2154. 10.1158/1078-0432.CCR-18-1449 30647082 10.1158/1078-0432.CCR-18-1449

[32] Yeghaian M, Tareco Bucho TM, De Bruin M, Schmitz A, Bodalal Z, Smit EF et al (2024) Can blood-based markers predict RECIST progression in non-small cell lung cancer treated with immunotherapy? J Cancer Res Clin Oncol 150(5):329. 10.1007/s00432-024-05814-2 38922374 10.1007/s00432-024-05814-2 PMC11208229

[33] Yoo SK, Fitzgerald CW, Cho BA, Fitzgerald BG, Han C, Koh ES et al (2025) Prediction of checkpoint inhibitor immunotherapy efficacy for cancer using routine blood tests and clinical data. Nat Med 31(3):869–880. 10.1038/s41591-024-03398-5 39762425 10.1038/s41591-024-03398-5 PMC11922749

[34] Huang H, Huang Z, Ge J, Yang J, Chen J, Xu B et al (2023) CD226 identifies functional CD8+T cells in the tumor microenvironment and predicts a better outcome for human gastric cancer. Front Immunol 14:1150803. 10.3389/fimmu.2023.1150803 37056782 10.3389/fimmu.2023.1150803 PMC10086426

[35] Nutsch K, Banta KL, Wu TD, Tran CW, Mittman S, Duong E et al (2024) TIGIT and PD-L1 co-blockade promotes clonal expansion of multipotent, non-exhausted antitumor T cells by facilitating co-stimulation. Nat Cancer 5(12):1834–1851. 10.1038/s43018-024-00870-6 39681653 10.1038/s43018-024-00870-6 PMC11663793

[36] Banta KL, Xu X, Chitre AS, Au-Yeung A, Takahashi C, O’Gorman WE et al (2022) Mechanistic convergence of the TIGIT and PD-1 inhibitory pathways necessitates co-blockade to optimize anti-tumor CD8+ T cell responses. Immunity 55(3):512-526.e9. 10.1016/j.immuni.2022.02.005 35263569 10.1016/j.immuni.2022.02.005 PMC9287124

