# Supplementary material for: Evaluation of the predictive and prognostic potential of blood immune cell profiles in metastatic cancer patients treated with immune checkpoint inhibitors
Source: Cancer Immunol Immunother. 2026 May 2;75(5):161. doi: 10.1007/s00262-026-04404-0 (PMC13135614; doi:10.1007/s00262-026-04404-0)
Supplement: Supplementary file 1 — Supplementary file1 (PDF 2548 KB) [file 262_2026_4404_MOESM1_ESM.pdf]

Supplemental Material

Supplemental Table 1. Flow staining protocol and the fluorescent monoclonal antibody panels used in the study.

Protocol	0.5-1 x10^6 cells were stained in each immunostaining per sample. Samples for panels 1-5 (Supplemental Table 1) were stained first with Zombie Aqua (Biolegend) viability stain, while samples for panel 6 were stained with Zombie Green (Biolegend) viability stain according to manufacturer's instructions to exclude the dead cells. Panel 1 samples were subjected to Fc receptor blocking (Human TruStain FcX, Biolegend) for 10 minutes according to manufacturer's instructions. The cells were then stained for 20 minutes in 0.5% BSA in PBS with each staining panel using fluorochrome labeled antibodies (Supplemental Table 1). To assess the PD-1 surface expression following PD-1 -blocking ICI therapy, an indirect staining approach was used. PE-conjugated secondary antibody specific for human IgG4 Fc (SouthernBiotech) was used to detect PD-1 blocking therapeutic antibodies [1,2]. After the staining, the samples were washed twice, resuspended in FACS buffer and acquired on a Novocyte Quanteon (Agilent) flow cytometer. All samples were analyzed with FlowJo Software (BD Biosciences). Flow cytometry results are presented as proportions (%) of parent populations as indicated in each plot.				
Staining panels	Antibody	Fluorochrome	Clone	Catalogue number	Manufacturer
Panel 1: Major immune cell subsets					
	TCR gamma-delta	BV421	B1	331217	Biolegend
	CD19	BV570	HIB19	302235	BioLegend
	CD56	BV605	HCD56	318333	BioLegend
	CD123	BV650	6H6	306019	BioLegend
	CD14	BV711	MφP9	563373	BD
	TCR Va7.2	BV785	3C10	351721	BioLegend
	CD11c	BB515	B-ly6	564491	BD
	CD27	BB700	M-T271	566450	BD
	TCR Va24-J18	PE	6B11	342903	Biolegend
	CD16	PE/Dazzle 594	3G8	302053	Biolegend
	HLA-DR	PE-Cy5	G46-6	562007	BD
	CD8	PE-Cy7	RPA-T8	560917	BD
	CD161	APC	HP-3G10	339911	Biolegend
	CD3	R718	UCTH1	566954	BD
	CD45	APC-F750	HI30	304061	Biolegend
Panel 2: B-cells and Treg					
	CD25	BB515	CD25	565096	BD
	CD38	BB700	CD38	566446	BD
	CD127	BV421	CD127	351309	Biolegend
	CD14	BV510	MΦP9	563079	BD
	CD16	BV510	3G8	302048	Biolegend
	CD56	BV510	HCD56	318340	Biolegend
	IgM	BV570	IgM	314517	Biolegend
	IgG	BV605	IgG	563246	BD
	CD3	BV650	CD3	563851	BD
	CD21	BV711	CD21	563163	BD
	IgD	BV785	IgD	348241	Biolegend

	CD4	APC	CD4	300514	Biolegend
	CD20	R718	CD20	566989	BD
	CD19	APC-F750	CD19	302258	Biolegend
	IgA	PE	IgA	130-114-002	Miltenyi
	CD27	PE-Dazzle594	CD27	356421	Biolegend
	CD10	PE-Cy5	CD10	312206	Biolegend
	CD45RA	PE-Cy7	CD45RA	304126	Biolegend
Panel 3:<br>T-cell subtypes					
	ICOS	BV605	C398.4A	313538	Biolegend
	CD14	BV510	MΦP9	563079	BD
	CD16		3G8	302048	Biolegend
	CD19		HIB19	302242	Biolegend
	CD56		HCD56	318340	Biolegend
	CD8	BV570	RPA-T8	301038	BioLegend
	CXCR3	BV421	IC6	562558	BD
	CCR7	BV650	G034H7	353233	BioLegend
	CD45RA	BV786	HI100	563870	BD
	CXCR5	BB515	RF8B2	564624	BD
	PD-1	PE	EH12.2H7	329906	Biolegend
	CrTh2	PE/Dazzle 594	BM16	350125	Biolegend
	CD161	PE-Cy5	DX12	551138	BD
	CD27	PE-Cy7	M-T271	356411	Biolegend
	CCR10	APC	REA326	130-120-406	Miltenyi
	CD3	R718	UCTH1	566954	BD
	CD4	APC-F750	RPA-T4	300560	BioLegend
	CCR4 (CD194)	BB700	1G1	566476	BD
	CCR6	BV711	G034E3	353435	Biolegend
Panels 4-5:<br>Co-inhibitory and co-stimulatory receptors (with/without anti-IgG4 PE)	TIGIT	BV421	A15153G	372709	Biolegend
	CD14	BV510	MΦP9	5630279	BD
	CD19		HIB19	302242	Biolegend
	CD8	BV570	RPA-T8	301038	BioLegend
	CD56	BV605	HCD56	318333	BioLegend
	CCR7	BV650	G034H7	353234	BioLegend
	TIM-3	BV711	F38-2E2	345023	Biolegend
	CD45RA	BV786	HI100	563870	BD
	CD226	BB515	DX11	565152	BD
	PD-1	PE	EH12.2H7	329906	Biolegend
	IgG4 Fc	PE		9200-09	Southern Biotech

	CD16	PE/Dazzle 594	3G8	302054	Biolegend
	LAG-3	PE-Cy5	11C3C65	369345	Biolegend
	CD27	BB700	M-T271	566450	BD
	CD160	A647	BY55	562362	BD Biosciences
	KLRG1	PE-Cy7	SA231A2	367719	Biolegend
	CD3	R718	M-T271	565116	BD
	CD4	APC-F750	RPA-T4	300560	Biolegend

References:

1. Huang A, Postow michael A, Orlowski RJ, mick R, Bengsch B, manne S, et al. T-cell invigoration to tumour burden ratio associated with anti-PD-1 response. Nat. 2017; 545:60–65. https://doi.org/10.1038/nature22079.
2. Zelba H, Bochem J, Pawelec G, Garbe C, Wistuba-Hamprecht K, Weide B. Accurate quantification of T-cells expressing PD-1 in patients on anti-PD-1 immunotherapy. Cancer Immunol Immunother. 2018;67(12):1845–51. https://link.springer.com/article/10.1007/s00262-018-2244-7

Supplemental Table 2. Radiological responses of retrospective and prospective cohorts.

	**Retrospective cohort**	**Prospective cohort**
**n**	**202**	**45**
**First response**		
Progressive disease (PD)	68 (33.7)	19 (42.2)
Stable Disease (SD)	47 (23.3)	10 (22.2)
Partial response (PR)	74 (36.6)	15 (33.3)
Complete response (CR)	12 (0.59)	1 (2.2)
Not evaluated	1 (0.5)	0 (0.0)
Non-responder group (PD+SD)	**115 (56.9)**	29 (64.4)
Responder group (PR+CR)	**86 (42.6)**	16 (35.6)

Supplemental Table 3. Cutoffs used for establishing prognostic groups for mPFS and mOS analyses in prospective data.

	mPFS	Events/records > vs. ≤ cut-off	mOS	Events/records > vs. ≤ cut-off
**Retrospective data, n=202**				
Leukocytes	11.2	20/22 vs. 142/179	8.1	50/69 vs. 80/132
Neutrophils	4.9	73/85 vs. 88/114	5.2	57/76 vs. 72/123
Monocytes	0.8	11/12 vs. 73/87	0.8	11/12 vs. 62/87
Lymphocytes	1.0	112/146 vs. 35/37	1.9	34/66 vs. 84/117
**Prospective data, n=45**				
Total monocytes	19.9	21/26 vs. 12/18	17.1	23/29 vs. 8/15
$CD14^+CD16^+$ monocytes	0.72	21/24 vs. 12/20	1.04	18/20 vs. 13/24
$CD14^+CD16^-$ classical monocytes	10.5	28/38 vs. 5/6	17.5	23/28 vs. 8/16
$CD16^+CD14^-$ nonclassical monocytes	0.89	19/20 vs. 14/24	0.63	22/26 vs. 9/18
cDCs	0.180	30/40 vs. 3/4	0.180	29/40 vs. 2/4
pDCs	0.09	28/35 vs. 5/9	0.11	25/33 vs. 6/11
Total lymphocytes	80.8	8/8 vs. 25/36	78.3	8/12 vs. 23/32
Total NK cells	19.5	5/5 vs. 28/39	8.33	25/36 vs. 6/8
Total $CD16^+$ NK cells	11.2	18/23 vs. 15/21	8.97	21/31 vs. 10/13
Total $CD56^+$ NK cells	0.09	30/36 vs. 3/8	0.09	29/36 vs. 2/8
T cells	62.0	5/5 vs. 28/39	58.2	6/10 vs. 25/34
B cells	8.66	7/7 vs. 26/37	1.65	27/40 vs. 4/4
$CD4^+CD27^+$	65.1	28/38 vs. 5/6	65.1	26/38 vs. 5/6
$CD4^+CD226^+$	95.6	6/10 vs. 27/34	95.5	8/12 vs. 23/32
$CD4^+KLRG1^+$	29.6	15/18 vs. 18/26	6.90	29/39 vs. 2/5
$CD4^+PD\text{-}1^+$	30.8	8/9 vs. 25/35	30.8	7/9 vs. 24/35
$CD4^+TIGIT^+$	32.2	3/6 vs. 30/38	30.2	4/7 vs. 27/37
$CD8^+CD27^+$	63.9	10/14 vs. 23/30	63.9	8/14 vs. 23/30
$CD8^+CD160^+$	22.0	7/7 vs. 26/37	10.0	21/27 vs. 10/17
$CD8^+CD226^+$	85.2	13/18 vs. 20/26	85.2	11/18 vs. 20/26
$CD8^+KLRG1^+$	58.3	28/39 vs. 5/5	85.5	8/15 vs. 23/29
$CD8^+PD\text{-}1^+$	14.3	22/31 vs. 11/13	24.9	12/16 vs. 19/28
$CD8^+TIGIT^+$	61.1	3/5 vs. 30/39	40.3	19/23 vs. 12/21

NK, Natural killer; cDCs, Classical dendritic cells; pDCs, Plasmacytoid dendritic cells

Supplemental Table 4. The association of pre-treatment counts/frequencies and fold changes of blood immune cells with radiological response, PFS and OS in the retrospective and flow* study cohorts of patients with NSCLC divided into **A)** total leukocytes, neutrophils and the subpopulations of the monocytic lineage, and **B)** total lymphocytes, NK, T and B cells.

A)

Endpoint			**Radiological early response**								**PFS**							**OS**						
Indicator			Pre-treatment median frequency/proportion (SD)				Median fold change* (SD)				HR (95% CI)			mPFS months				HR (95% CI)			mOS months			
Grouping criterion			R responder<br>NR non-responder				R responder<br>NR non-responder							> cell frequency/<br>proportion higher than cutoff<br>< lower than cutoff							> cell frequency/<br>proportion higher than cutoff<br>< lower than cutoff			
Group			R	NR	*p*	*FDR*	R	NR	*p*	*FDR*		*p*	*FDR*	>	<	*p*	*FDR*		*p*	*FDR*	>	<	*p*	*FDR*
Cell population	LEUCOCYTES	Leukocytes (E9/L)	7.30 (4.14)	8.05 (3.99)	0.25	0.67	0.97 (0.24)	0.90 (0.40)	0.75	0.88	**2.13 (1.05–4.31)**	**0.04**	**0.32**	**2.0 (1.9–2.1)**	**5.5 (3.7–7.3)**	**0.03**	**0.15**	**1.70 (1.04–2.83)**	**0.04**	**0.13**	**15.4 (3.6–27.2)**	**24.5 (12.7–36.4)**	**0.04**	**0.06**
	NEUTROPHILS	Neutrophils (E9/L)	4.70 (4.29)	5.50 (2.72)	0.13	0.64	0.96 (0.35)	0.84 (0.53)	0.77	0.88	1.50 (0.96–2.35)	0.08	0.32	5.2 (3.8–6.7)	7.7 (4.4–10.9)	0.07	0.18	**1.77 (1.07–2.94)**	**0.03**	**0.13**	**12.9 (3.0–22.9)**	**31.2 (19.0–43.4)**	**0.03**	**0.06**
	MONOCYTIC LINEAGE	Monocytes (E9/L)	0.50 (0.23)	0.55 (0.31)	0.41	0.73	1.00 (0.41)	1.00 (0.26)	0.63	0.84	2.20 (0.89–5.43)	0.09	0.32	**N/A**	**N/A**	**N/A**	N/A	**3.15 (1.33–7.45)**	**0.009**	**0.13**	**8.0 (6.8–9.2)**	**21.7 (17.9–25.5)**	**0.006**	**0.04**
		Monocytes*	23.03 (11.48)	32.64 (13.37)	0.14	0.64	1.14 (0.78)	0.98 (0.48)	0.53	0.84	2.88 (0.82–10.1)	0.10	0.32	N/A	N/A	N/A	N/A	6.75 (0.86–53.26)	0.07	0.13	N/A	N/A	N/A	N/A
		Classical monocytes*	19.55 (11.02)	31.65 (12.48)	0.12	0.64	1.12 (0.85)	0.94 (0.52)	0.49	0.84	1.63 (0.21–12.63)	0.64	0.73	N/A	N/A	N/A	N/A	8.23 (1.06–63.94)	0.04	0.13	N/A	N/A	N/A	N/A
		Non-classical monocytes*	0.71 (0.70)	0.48 (0.90)	0.77	0.93	1.02 (4.49)	0.64 (1.42)	0.09	0.80	1.53 (0.59–3.99)	0.39	0.57	7.7 (0.0–23.4)	12.2 (3.8–20.7)	0.38	0.48	0.89 (0.33–2.40)	0.82	0.82	26.8 (15.2–38.5)	23.3 (15.1–31.5)	0.82	0.82
		Intermediate monocytes*	1.08 (0.85)	1.12 (1.17)	0.56	0.90	1.43 (11.37)	0.82 (3.82)	0.12	0.80	2.30 (0.74–7.08)	0.15	0.34	N/A	N/A	N/A	N/A	3.14 (0.99–9.99)	0.052	0.13	**18.6 (2.6–34.5)**	**26.4 (10.1–42.7)**	**0.04**	**0.06**
		Dendritic cells cDCs*	0.55 (3.61)	0.39 (0.44)	0.39	0.73	0.95 (1.23)	1.52 (5.87)	0.45	0.84	4.40 (0.58–33.6)	0.15	0.34	N/A	N/A	N/A	N/A	6.36 (0.80–50.86)	0.08	0.13	N/A	N/A	N/A	N/A
		Dendritic cells pDCs*	0.25 (0.16)	0.17 (0.15)	0.99	0.99	1.09 (0.51)	0.94 (0.73)	0.37	0.84	1.98 (0.57–6.93)	0.28	0.45	N/A	N/A	N/A	N/A	1.71 (0.54–5.44)	0.36	0.44	N/A	N/A	N/A	N/A

*prospective flow cytometry cohort; FDR, FDR-adjusted p-value

B)

Endpoint			**Radiological response**								**PFS**							**OS**						
Indicator			Pre-treatment median frequency (SD)				Median fold change* (SD)				HR (95% CI)			mPFS months				HR (95% CI)			mOS months			
Grouping criterion			R responder<br>NR non-responder				R responder<br>NR non-responder							> cell frequency/<br>proportion higher than cutoff<br><lower than cutoff							> cell frequency/<br>proportion higher than cutoff<br>< lower than cutoff			
Group			R	NR	*p*	*FDR*	R	NR	*p*	*FDR*		*p*	*FDR*	>	<	*p*	*FDR*		*p*	*FDR*	>	<	*p*	*FDR*
Cell population	LYMPHO-CYTES	Lymphocytes (E9/L)	1.65 (0.74)	1.80 (0.81)	0.73	0.93	1.00 (0.17)	1.00 (0.22)	0.93	0.93	0.70 (0.41–1.19)	0.18	0.36	5.5 (3.5–7.6)	4.6 (4.5–4.6)	0.18	0.30	0.92 (0.54–1.57)	0.40	0.46	20.9 (17.9–23.9)	18.6 (8.1–29.0)	0.76	0.82
		Lymphocytes*	70.97 (10.25)	61.87 (14.21)	0.38	0.73	1.04 (0.25)	0.95 (0.27)	0.45	0.84	1.02 (0.23–4.50)	0.98	0.98	N/A	N/A	N/A	N/A	0.17 (0.02–1.33)	0.09	0.13	N/A	N/A	N/A	N/A
	NK CELLS	Total NK cells*	10.85 (8.34)	12.55 (9.85)	0.93	0.99	1.04 (0.94)	0.99 (1.13)	0.61	0.84	2.73 (0.83–9.02)	0.10	0.32	N/A	N/A	N/A	N/A	**0.12 (0.02–0.66)**	**0.02**	**0.13**	N/A	N/A	N/A	N/A
		CD16+ NK cells*	9.37 (8.96)	11.25 (9.16)	0.81	0.93	1.01 (64.2)	0.97 (1.89)	0.53	0.84	1.39 (0.54–3.63)	0.50	0.62	7.4 (0.0–22.1)	14.3 (0.0–31.2)	0.49	0.49	0.37 (0.12–1.10)	0.07	0.13	N/A	N/A	N/A	N/A
		CD56+ NK cells*	0.14 (0.28)	0.26 (0.25)	0.21	0.67	1.07 (0.90)	0.71 (0.46)	0.15	0.80	2.34 (0.53–10.32)	0.26	0.45	N/A	N/A	N/A	N/A	4.37 (0.57–33.32)	0.16	0.21	N/A	N/A	N/A	N/A
	T CELLS*		53.35 (7.77)	43.25 (12.47)	0.16	0.64	0.91 (0.23)	0.92 (0.26)	0.93	0.93	0.82 (0.11–6.33)	0.85	0.91	N/A	N/A	N/A	N/A	0.67 (0.15–2.97)	0.60	0.64	N/A	N/A	N/A	N/A
	B CELLS*		3.52 (2.75)	3.72 (3.50)	0.80	0.93	0.90 (0.65)	0.73 (0.66)	0.45	0.84	1.62 (0.45–5.83)	0.46	0.61	N/A	N/A	N/A	N/A	0.32 (0.09–1.21)	0.09	0.13	N/A	N/A	N/A	N/A

*prospective flow cytometry cohort; FDR, FDR-adjusted p-value; NK, Natural killer

Supplemental Table 5. Main immune cell subset frequencies in ICI responders and non-responders.

**Cell subsets**	Responder	Non-responder		Responder	Non-responder	
	**Median**		***p***	**Fold-change median**		***p***
Naïve $CD19^+$ B ($CD27^-IgD^+$)	66,3	73,3	0,4964	1,051	1,008	0,1756
Unswitched memory $CD19^+$ B ($CD27^+IgD^+$)	5,69	4,32	0,8881	1,067	0,8986	**0,0279**
Switched memory $CD19^+$ B ($CD27^+IgD^-$)	15	16,2	0,9464	0,9593	0,9956	0,7178
Memory $CD19^+$ B ($CD27^-IgD^-$)	7,00	4,76	**0,0497**	0,9073	1,057	0,0715
$CD4^+$ T cells	61,5	66,2	0,4007	0,9901	0,9531	0,553
Naïve $CD4^+$ T ($CD45RA^+CCR7^+CD27^+$)	33,0	34,2	0,2556	0,9957	0,9539	0,6332
Central memory $CD4^+$ T ($CD45RA^-CCR7^+CD27^+$)	29,8	34,4	0,2025	0,9723	0,9593	0,6127
$CD27^+$ effector memory $CD4^+$ T ($CD45RA^-CCR7^-CD27^+$)	14,9	12,5	0,2453	1,092	1,098	0,8510
$CD27^-$ effector memory $CD4^+$ T ($CD45RA^-CCR7^-CD27^-$)	10,5	9,65	0,2556	0,985	1,181	0,5148
TEMRA $CD4^+$ T ($CD45RA^+CCR7^-CD27^-$)	2,28	2,92	0,8209	1,062	1,056	0,9479
Total Tregs ($CD4^+CD25^+CD127lo$)	6,84	5,18	0,2279	1,139	1,008	0,1254
Naive Tregs	45,6	41,3	>0,9999	0,9926	0,9737	0,7395
Memory Tregs	53,8	57,6	0,9655	1,013	1,042	0,4962
$CD8^+$ T cells	32,0	27,3	0,4583	1,031	1,086	0,7395
Naïve $CD8^+$ T ($CD45RA^+CCR7^+CD27^+$)	5,66	12,6	0,1244	0,8873	0,788	0,851
CM $CD8^+$ T ($CD45RA^-CCR7^+CD27^+$)	7,79	6,10	0,8448	0,8692	0,7782	0,9885
$CD27^+$ effector memory $CD8^+$ T ($CD45RA^-CCR7^-CD27^+$)	23,0	19,6	0,8114	0,9648	1,021	0,5337
$CD27^-$ effector memory $CD8^+$ T ($CD45RA^-CCR7^-CD27^-$)	8,59	9,26	0,3169	1,067	1,010	0,9194
TEMRA $CD8^+$ T ($CD45RA^+CCR7^-CD27^-$)	25,3	29,3	0,6031	1,080	1,115	0,9885

Supplemental Table 6. Frequencies of immune cell subsets pre and post ICI regardless of the response groups.

Cell subsets	Pre-ICI	Post-ICI	
	**Median**	**Median**	**p-value**
Classical monocytes ($CD14^+CD16^-$)	20,8	21,5	0,5783
Intermediate monocytes ($CD14^+CD16^+$)	0,78	1,16	0,1531
Non-classical monocytes ($CD14^-$ $CD16^+$)	0,89	0,9	0,5684
Classical dendritic cells (cDC) ($CD11c^+CD14^-CD16^-$)	0,47	0,59	0,6906
Plasmacytoid dendritic cells (pDCs) ($CD123^+CD11c^-$)	0,21	0,18	**0,0401**
Total NK cells	12,5	13,5	0,3592
$CD16^+$ NK cells	11,2	12	0,4205
$CD56^+$ NK cells	0,23	0,22	0,2903
Total $CD19^+$ B cells	7,27	6,3	0,2316
Naïve $CD19^+$ B ($CD27^-IgD^+$)	69,2	70	0,3037
Unswitched memory $CD19^+$ B ($CD27^+IgD^+$)	4,15	4,01	0,3137
Switched memory $CD19^+$ B ($CD27^+IgD^-$)	16,2	16,4	0,5273
DN Memory $CD19^+$ B ($CD27^-$ IgD^-)	5,64	5,35	0,6712
Total $CD3^+$ T cells	52	50,6	0,1614
Total $CD4^+$ T cells	66,2	63,6	**0,0331**
Central memory $CD4^+$ T ($CD45RA^-CCR7^+CD27^+$)	31,4	33	0,1131
$CD27^+$ effector memory $CD4^+$ T ($CD45RA^-CCR7^-CD27^+$)	13,3	14,8	**0,0184**
CD27- effector memory $CD4^+$ T ($CD45RA^-CCR7^-CD27^-$)	9,65	11,1	0,26
TEMRA $CD4^+$ T ($CD27^-CD45RA^+CCR7^-$)	2,68	2,94	0,3518
Total Tregs ($CD4^+CD25^+CD127lo$)	5,54	6,01	0,3853
Naive Tregs	44,4	46,1	0,2455
Memory Tregs	54,5	52,9	0,2261
Total $CD8^+$ T cells	27,3	29	0,1978
Naïve $CD8^+$ T	7,23	6,85	**0,0024**

(CD45RA^{+}CCR7^{+}CD27^{+})			
CM CD8^{+} T (CD45RA^{-}CCR7^{+}CD27^{+})	7,28	5,66	0,0585
CD27^{+} effector memory CD8^{+} T (CD45RA^{-}CCR7^{-}CD27^{+})	19,4	18,7	0,6661
CD27^{-} effector memory CD8^{+} T (CD45RA^{-}CCR7^{-}CD27^{-})	8,73	9,69	0,6864
TEMRA CD8^{+} T (CD27^{-}CD45RA^{+}CCR7^{-})	25,3	28	**0,0046**
CD8^{+}PD-1^{+} memory T	17,4	9,3	**<0,0001**
CD8^{+}TIGIT^{+} memory T	40,3	44,5	**0,015**
CD8^{+}KLRG1^{+} memory T	78	79,8	0,0666
CD8^{+}CD160^{+} memory T	14,4	14,4	0,2455
CD8^{+}CD27^{+} memory T	57,3	54,5	**0,0065**
CD8^{+}CD226^{+} memory T	84	81,5	0,2883
CD8^{+}LAG-3^{+} memory T	0,068	0,14	N/A
CD8^{+}TIM-3^{+} memory T	0,02	0,04	N/A
CD4^{+}PD-1^{+} memory T	22,8	13,2	**<0,0001**
CD4^{+}TIGIT^{+} memory T	24,6	25,9	**0,002**
CD4^{+}KLRG1^{+} memory T	25,4	25,2	0,2571
CD4^{+}CD160^{+} memory T	0,084	0,15	N/A
CD4^{+}CD27^{+} memory T	78,2	79,2	0,1052
CD4^{+}CD226^{+} memory T	93,5	94	0,8101
CD4^{+}LAG-3^{+} memory T	0,02	0,08	N/A
CD4^{+}TIM-3^{+} memory T	0,02	0,03	N/A

Supplemental Table 7. Frequencies, fold change, mPFS and mOS of co-inhibitory and co-activation surface receptor expressing memory $CD4^{+}$ and $CD8^{+}$ T cells in prospective (flow cytometry) study cohort.

Endpoint			Radiological early response								PFS							OS						
Indicator			Pre-treatment median frequency (SD)				Median fold change* (SD)				HR (95% CI)			mPFS months				HR (95% CI)			mOS months			
Grouping criterion							Radiological response<br>R responder<br>NR non-responder							> cell frequency/ proportion higher than cutoff<br>< lower than cutoff							> cell frequency/ proportion higher than cutoff<br>< lower than cutoff			
Group			R	NR	*p*	*FDR*	R	NR	*p*	*FDR*		*p*	*FDR*	>	<	*p*	*FDR*		*p*	*FDR*	>	<	*p*	*FDR*
Cell population	CD4+ T CELLS	CD27	76.2 (18.4)	78.5 (12.6)	0.34	0.75	1.00 (0.07)	0.99 (0.12)	0.92	0.92	1.00 (0.97–1.02)	0.86	0.99	N/A	N/A	N/A	N/A	1.00 (0.97–1.02)	0.75	0.99	N/A	N/A	N/A	N/A
		CD226	93.0 (2.48)	94.5 (7.25)	0.60	0.83	1.00 (0.02)	1.00 (0.13)	0.83	0.92	0.97 (0.91–1.02)	0.21	0.61	**16.6 (12.8–N/A)**	**2.7 (1.9–12.4)**	**0.05**	0.13	1.02 (0.95–1.10)	0.54	0.99	26.8 (24.3–N/A)	13.6 (7.2–23.3)	0.06	0.14
		KLRG-1	24.4 (18.47)	27.1 (17.20)	0.94	0.94	1.04 (0.38)	1.06 (1.10)	0.43	0.92	1.01 (0.99–1.04)	0.22	0.61	**2.0 (1.4–N/A)**	**13.2 (3.8–N/A)**	**0.01**	**0.05**	1.01 (0.99–1.03)	0.41	0.99	N/A	N/A	N/A	N/A
		PD-1	25.2 (12.34)	21.2 (8.85)	0.28	0.75	0.62 (0.17)	0.53 (0.31)	0.46	0.92	1.01 (0.97–1.04)	0.71	0.99	N/A	N/A	N/A	N/A	1.01 (0.97–1.04)	0.67	0.99	N/A	N/A	N/A	N/A
		TIGIT	24.7 (10.98)	23.2 (6.46)	0.24	0.75	1.11 (0.27)	1.14 (0.32)	0.48	0.92	**0.96 (0.92–1.00)**	**0.04**	0.44	N/A	N/A	N/A	N/A	0.97 (0.93–1.01)	0.16	0.99	N/A	N/A	N/A	N/A
	CD8+ T CELLS	CD27	63.6 (23.81)	58.2 (16.71)	0.94	0.94	0.93 (0.15)	0.93 (0.18)	0.85	0.92	1.00 (0.98–1.02)	0.82	0.99	14.3 (7.7–N/A)	2.8 (2.0–12.4)	0.09	0.14	1.00 (0.98–1.02)	0.95	0.99	31.7 (20.9–N/A)	13.6 (5.5–26.4)	0.10	0.14
		CD160	12.4 (9.28)	14.3 (9.15)	0.42	0.75	1.18 (0.69)	1.01 (0.35)	0.14	0.92	1.03 (0.99–1.08)	0.11	0.61	N/A	N/A	N/A	N/A	1.02 (0.97–1.06)	0.43	0.99	15.6 (5.4–N/A)	24.3 (14.3–N/A)	0.10	0.14
		CD226	**89.6 (12.20)**	**83.0 (10.63)**	**0.04**	0.44	1.00 (0.08)	0.99 (0.25)	0.61	0.92	0.99 (0.96–1.02)	0.55	0.99	12.8 (7.4–N/A)	2.7 (1.9–13.2)	0.11	0.14	1.00 (0.97–1.03)	0.96	0.99	**26.8 (24.3–N/A)**	**13.1 (5.5–23.3)**	**0.02**	0.14
		KLRG-1	71.3 (19.05)	81.1 (14.73)	0.92	0.94	1.01 (0.09)	1.01 (0.18)	0.85	0.92	1.00 (0.98–1.02)	0.96	0.99	N/A	N/A	N/A	N/A	1.00 (0.98–1.02)	0.99	0.99	34.3 5.5–N/A)	20.4 (13.6–26.8)	0.21	0.21
		PD-1	17.9 (12.72)	21.7 (10.90)	0.48	0.75	0.57 (0.19)	0.50 (0.38)	0.55	0.92	1.00 (0.97–1.03)	0.99	0.99	7.4 (2.7–N/A)	3.8 (1.6–N/A)	0.15	0.15	1.01 (0.97–1.04)	0.66	0.99	12.0 (5.2–N/A)	23.3 (14.3–N/A)	0.20	0.21
		TIGIT	35.3 (18.65)	44.3 (16.59)	0.38	0.75	1.06 (0.46)	1.04 (0.96)	0.50	0.92	1.00 (0.98–1.02)	0.71	0.99	N/A	N/A	N/A	N/A	1.00 (0.98–1.03)	0.73	0.99	15.6 (5.5–N/A)	24.3 (14.3–N/A)	0.09	0.14

FDR, FDR-adjusted p-value

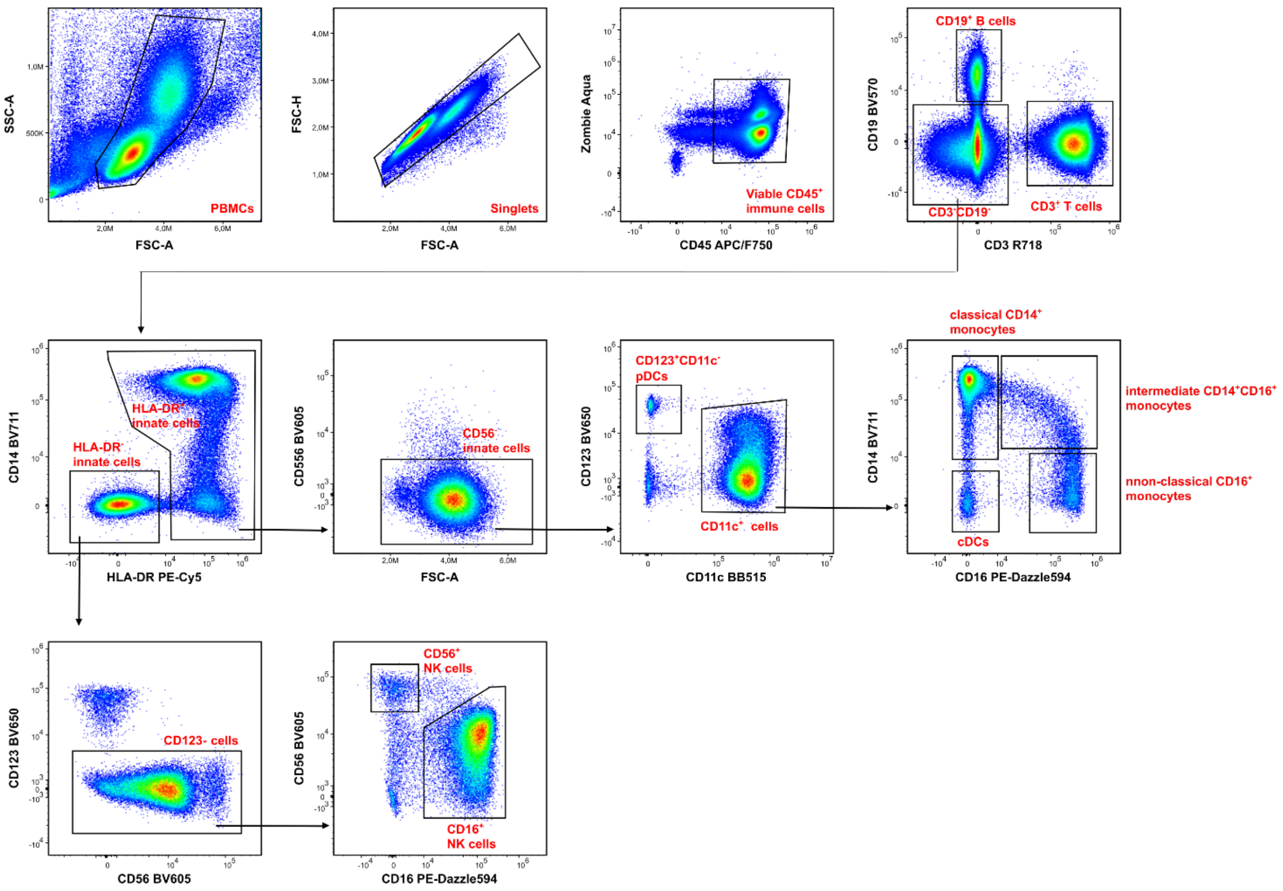

Supplemental Fig. 1 Representative gating of major immune cell subsets. First, PBMCs were gated according to FSC-A and SSC-A characteristics, and singlets were gated using FSC-A and FSC-H. Next, viable (Zombie Aqua-) CD45^{+} immune cells were gated. To distinguish B (CD19^{+}), T (CD3^{+}) and non-T/B (CD3^{-}CD19^{-}) cells, a combination of CD3 and CD19 were used. To delineate innate cells, CD3^{-}CD19^{-} non-T/B cells were gated into HLA-DR^{+} and HLA-DR^{-} compartments. HLA-DR^{+}CD56^{-} cells were further separated into CD123^{+}CD11c^{-} pDCs and CD11c^{+} cells consisting of classical CD14^{+} monocytes, CD14^{+}CD16^{+} intermediate monocytes and non-classical CD16^{+} monocytes. Cells that were CD11c^{+}CD14^{-}CD16^{-} were labeled as cDCs. Total NK cells were distinguished as HLA-DR^{-}CD123^{-}CD56^{+}CD16^{+}, and further delineated into CD56^{+} NK cells, and CD16^{+}CD56lo NK cells

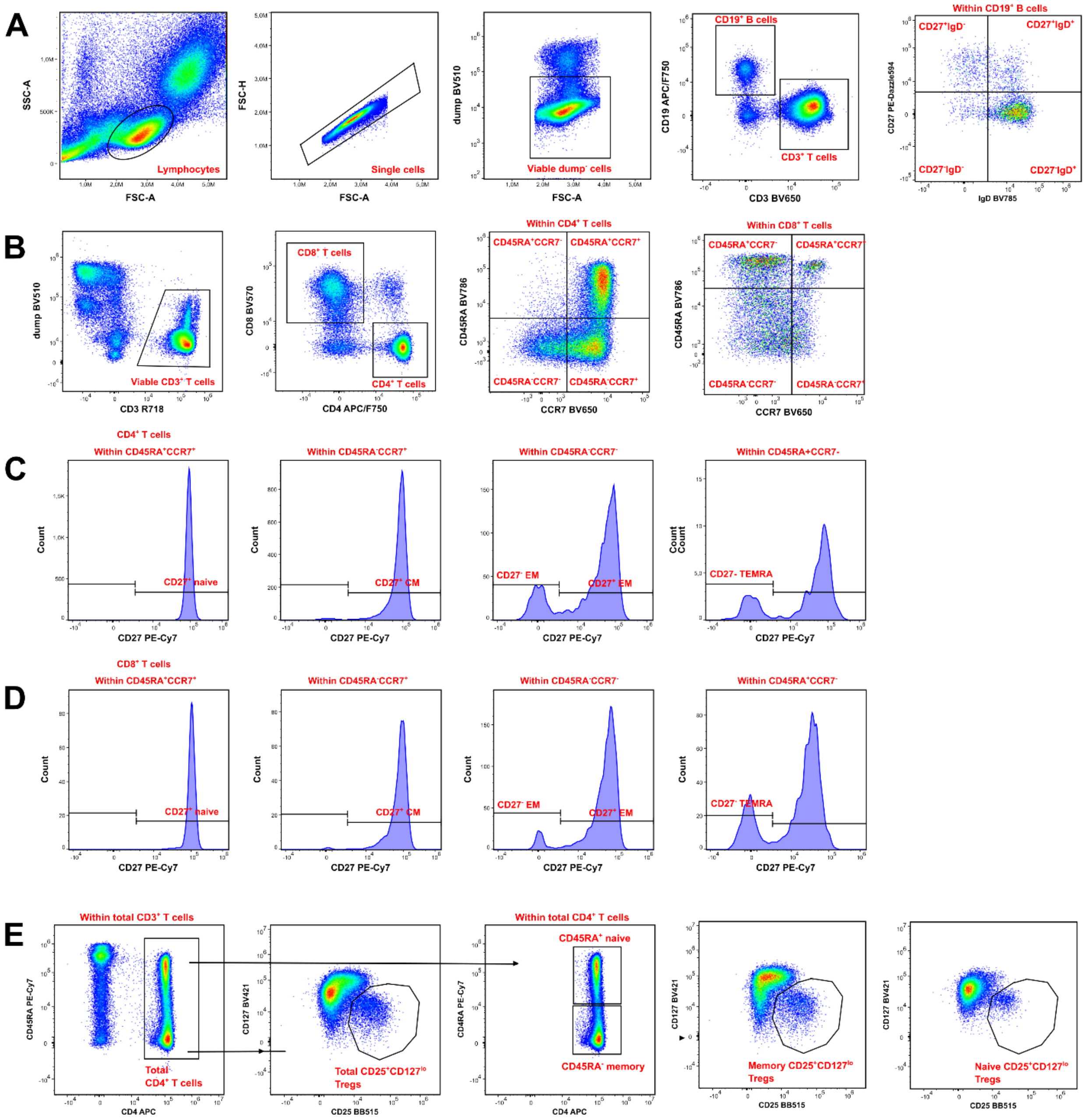

Supplemental Fig. 2 Representative gating of B and T-cell subsets. Lymphocytes were gated according to morphohological properties using FSC-A and SSC-A, and singlets were gated using FSC-A and FSC-A. Viable (Zombie Aqua-dump-) lymphocytes were gated, and further delineated into CD19$^+$ B and CD3$^+$ T cells. CD19$^+$ B cells were gated into naive and memory subsets according to CD27+/-IgD+/- where CD27-IgD+ represented naive B cells, CD27$^+$IgD$^+$ unswitched memory B cells, CD27$^+$IgD$^-$ switched memory B cells and CD27$^-$IgD$^-$ double-negative (DN) memory B cells. (A) Viable (Zombie Aqua-dump-) CD3$^+$ T cells were gated, and further delineated into CD4$^+$ and CD8$^+$ T cells. (B) Naive and memory subsets were defined according to CD45RA+/-CCR7$^-$. Naive cells were defined as CD45RA$^+$CCR7$^+$CD27$^+$, central memory (CM) as CD45RA$^-$CCR7$^+$CD27$^+$, effector memory (EM) as CD45RA$^-$CCR7$^-$CD27+/- and terminal effector memory (TEMRA) as CD45RA$^+$CCR7$^-$CD27$^-$ within both CD4$^+$ (C) and CD8$^+$ T cells (D). Total Tregs were gated from CD4$^+$ T cells and defined as CD25$^+$CD127lo. CD4$^+$ T cells were gated into naive (CD45RA$^+$) and memory (CD45RA$^-$) subsets and memory Tregs defined as CD45RA$^-$CD25$^+$CD127lo and naive Tregs as CD45RA$^+$CD25$^+$CD127lo (E)

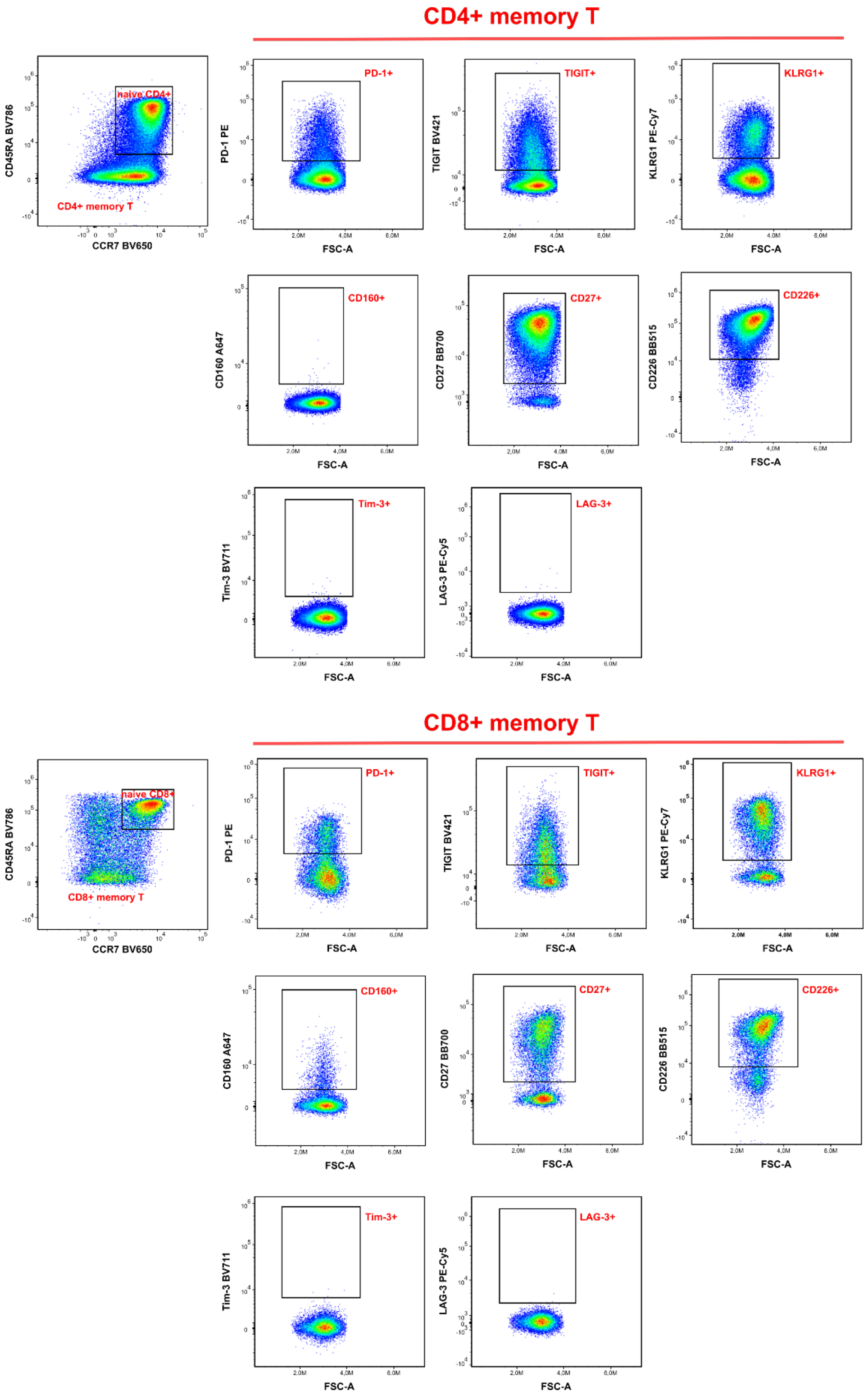

Supplemental Fig. 3 Representative gating for co-inhibitory and co-activating cell surface receptors on $CD4^+$ and $CD8^+$ T cells. Lymphocytes were gated according to morphological properties using FSC-A and SSC-A, and singlets were gated using FSC-A and FSC-A, then delineated into viable $CD3^+$ T cells and subsequently total $CD4^+$ and $CD8^+$ T cells were gated as shown in Supplemental Fig. 2. $CD4^+$ and $CD8^+$ T cells were gated into naïve ($CD45RA^+CCR7^+$) and total memory cells using Boolean NOT-operator (i.e. total memory is all cells that do not fall into the naïve gate). Subsequently the expression of co-inhibitory and co-activation cell surface markers PD-1, TIGIT, KLRG1, CD160, CD27, CD226, Tim-3 and LAG-3 was assessed in total $CD4^+$ and $CD8^+$ memory T cell compartments

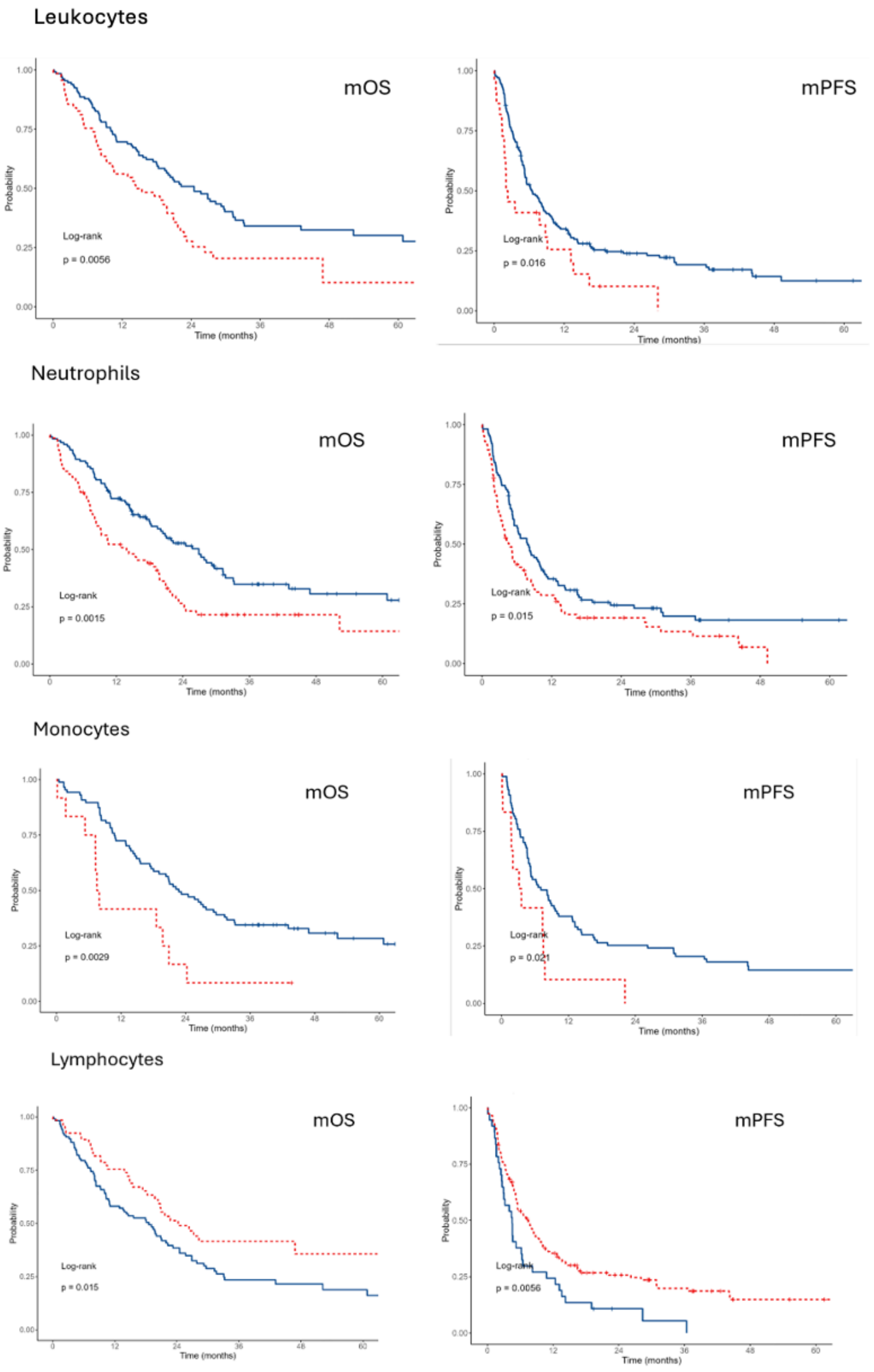

Supplemental Fig. 4 Overall and progression free survival of patients in the retrospective cohort divided into two survival groups based on pre-treatment leucocyte, neutrophil, monocyte and lymphocyte levels

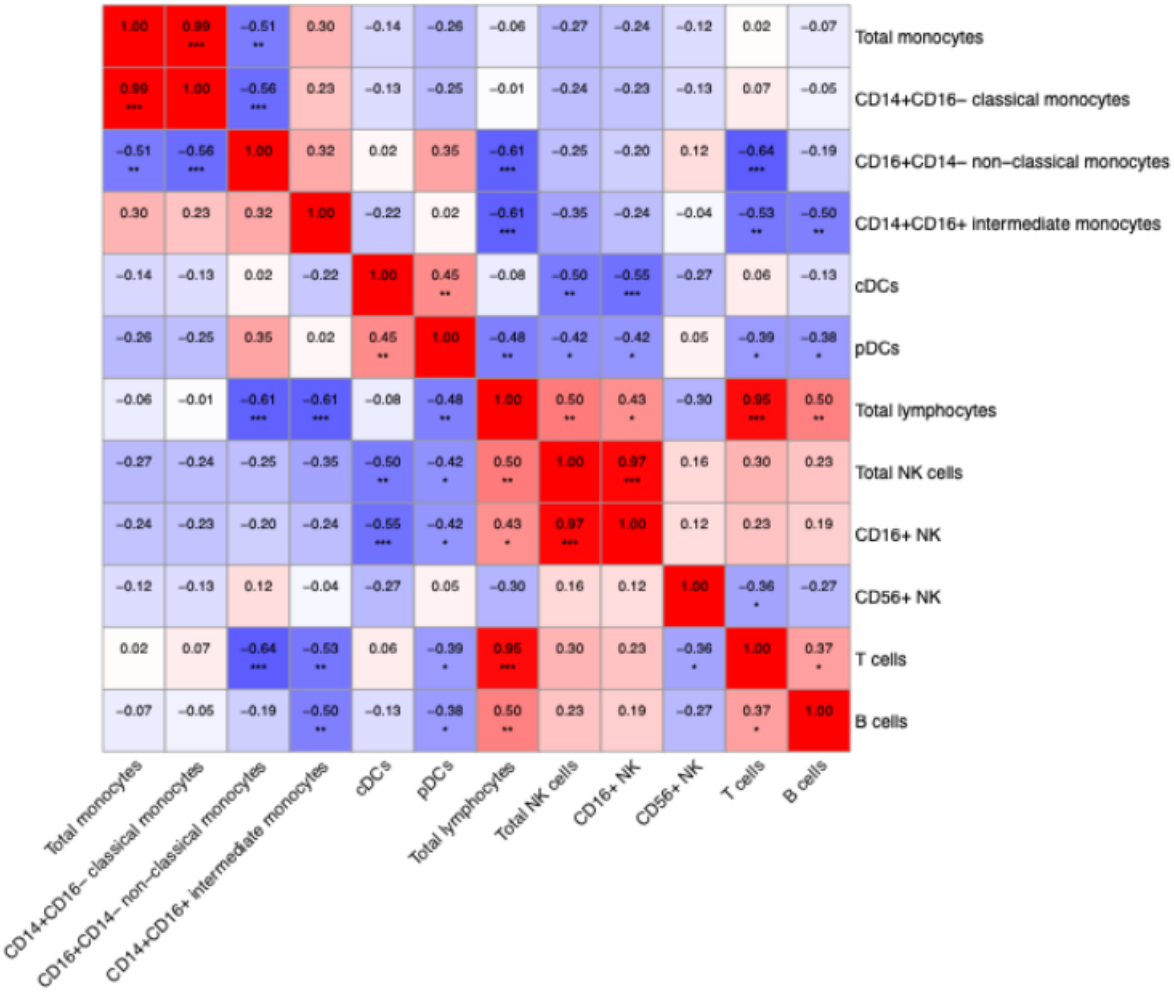

Supplemental Fig. 5 Pairwise correlations of the major immune cell subsets studied

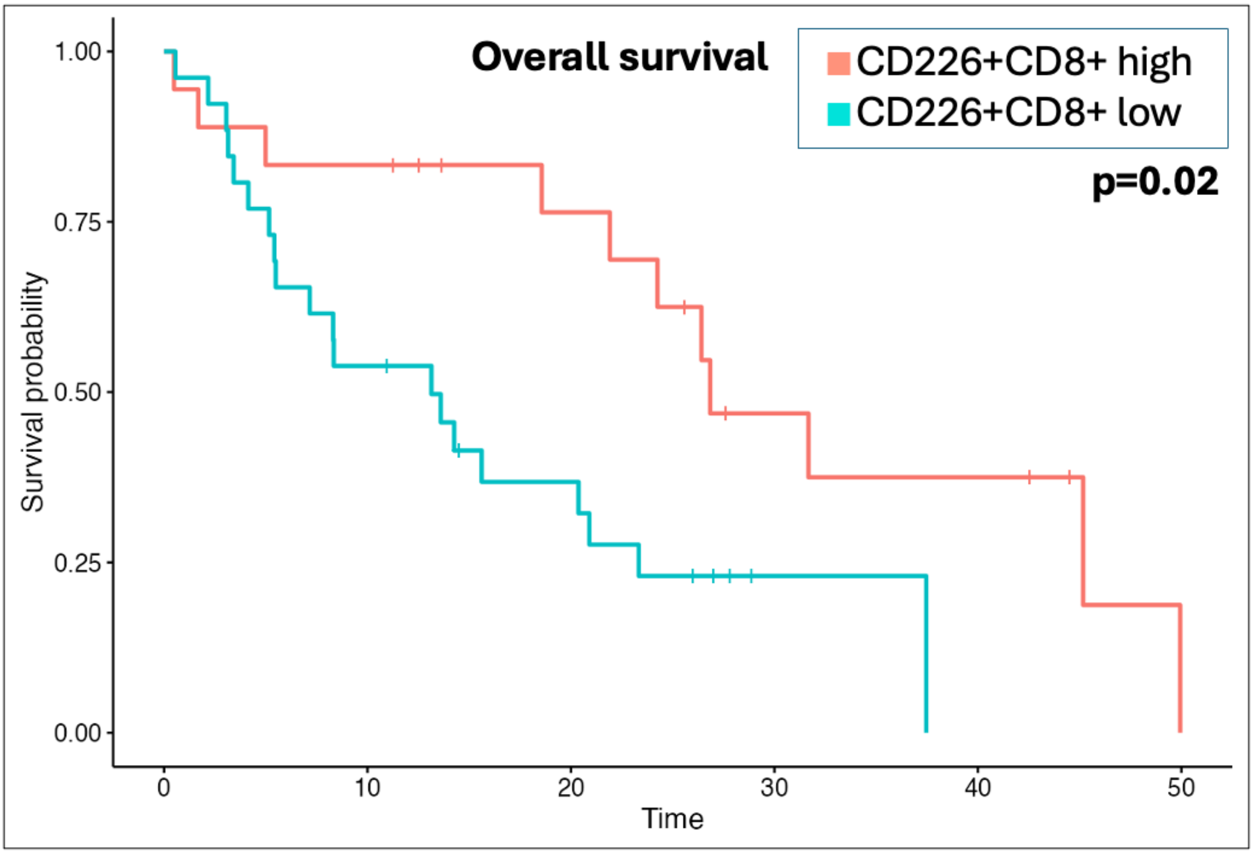

Supplemental Fig. 6 Overall survival of patients with high and low CD226^{+}CD8^{+}
